# Eggshell-Derived Copper Calcium Hydroxy Double Salts and Their Activity for Treatment of Highly Polluted Wastewater

**DOI:** 10.1021/acsomega.3c05758

**Published:** 2023-11-29

**Authors:** Yiping Han, Jirawat Trakulmututa, Taweechai Amornsakchai, Supakorn Boonyuen, Nicha Prigyai, Siwaporn Meejoo Smith

**Affiliations:** † Natural Resources and Waste Module, Department of Chemistry, Faculty of Science, Mahidol University, Rama VI Rd, Rajathewi 10400, Thailand; ‡ Center of Sustainable Energy and Green Materials and Department of Chemistry, Faculty of Science, Mahidol University, Salaya, Nakorn Pathom 73170, Thailand; § Department of Chemistry, Faculty of Science and Technology, Thammasat University, Paholyothin, Klong-Luang, Pathumthani 12120, Thailand

## Abstract

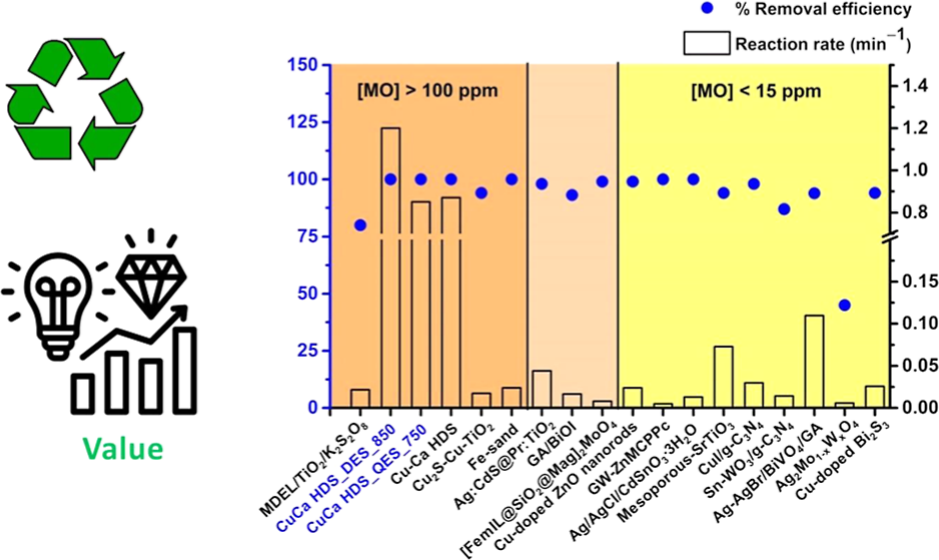

By using methyl orange (MO) removal as a model reaction, the best temperatures for processing eggshells are 750 °C and above to obtain biobased CaO materials, a raw material for producing CuCa hydroxy double salt (HDS) materials with high efficiency in treatments of highly polluted wastewater (the initial concentration of MO is 500 ppm). Characterization techniques employed in this study include power X-ray diffraction, scanning electron microscopy, thermogravimetric analysis, nitrogen adsorption–desorption analysis, and the colorimetric method, as well as energy-dispersive X-ray, infrared-, and electron spin resonance spectroscopies. Complete MO removal and high chemical oxygen demand (COD) efficiencies (>90%) can be achieved after 3 min treatments of the aqueous MO with the calcined eggshell-derived CuCa HDS materials. The spent, deactivated HDS materials can be regenerated by an acid wash method. The activity of CuCa HDS materials in MO removal is unaffected by eggshell sources, implying that sorting steps may be unnecessary when eggshell food waste (duck, quail, and hen eggshells) is collected to produce biobased CaO. The findings of this study demonstrated that eggshells can be used in place of limestone and could be a more sustainable, renewable, and cost-effective source for material development and other applications.

## 1 Introduction

Calcium oxide (CaO), commonly referred to as quicklime, is extracted from nature through the mining, crushing, and burning of limestone. This process involves heating limestone at high temperatures, causing it to undergo a chemical reaction that releases carbon dioxide and leaves behind CaO,^1−3^ which is widely used in various industries, including construction, agriculture, and water treatment. On the other hand, CaO can instead be extracted from renewable resources such as mollusk shells and eggshells, typical waste from food processing. The chemical composition of eggshells is composed of calcium carbonate (94%), calcium phosphate (1%), magnesium carbonate (1%), and organic matter (4%).^4^ Based on the literature, the properties of eggshell-derived CaO are similar to those extracted from limestones.^5,6^ Nonetheless, large quantities of eggshell waste have frequently been utilized in low-tech applications such as fertilizers, soil pH adjustment components, animal feeds, and compost.^7^ Although eggshell waste could be used to produce high-value calcium supplements, rigorous testing for contaminants and proper processing techniques will be required to ensure the purity and effectiveness of the eggshell-waste-derived calcium supplements as well as the health and safety of consumers. Thus, nonfood applications may be more feasible, but research is needed to explore potential uses for eggshell waste in new and innovative ways, such as in the area of material development, to maximize its value and reduce waste. Similarly, burning processes are needed to convert limestone to quicklime as well as to convert eggshells to biobased CaO. Nevertheless, utilizing eggshell waste not only reduces the environmental impact of mining and burning limestone but also provides a sustainable solution for industries, which should lead to a more circular economy and a reduced reliance on traditional, nonrenewable calcium sources.

Burning mollusk shells and eggshells, causing the conversion of CaCO_3_ to CaO, has been extensively performed to explore its applications in the development of construction materials such as cement.^8,9^ Several research groups produce a low-cost heterogeneous catalyst for biodiesel production^10,11^ or sorbents for CO_2_,^12,13^ metal ions,^14,15^ and radionuclides.^16,17^ Henrique and co-workers^18^ utilized a mollusk shell-derived CaO to synthesize a high-value CuAl-layered double hydroxide (CuAl LDH) material. Such a metal oxide-assisted method is one of the simple approaches to prepare LDH materials via ion exchange reactions in an aqueous solution. Likewise, copper-based hydroxy double salt (HDS) materials, classed in the LDH family, contain Cu(II) and another metal ion in the brucite-like layered structure. By varying metal oxide precursors, i.e., CaO, ZnO, La_2_O_3_, or PbO, respectively, to react with aqueous copper salt (such as copper nitrate), one will simply produce the CuCa, CuZn, CuLa, and CuPb HDS materials, which are promising materials for effective aqueous methyl orange (MO) dye removal via oxidative reactions (wet air oxidation) under ambient conditions without the requirement of light irritation or additional oxidant.^19,20^ More recently, the effective removal of dyes from water in the presence of Cu-based HDS materials was described by simultaneous processes, i.e., adsorption, oxidation, and intercalation reactions.^38^ Although the Cu-based HDS materials did accelerate the dye removal, they should not be called catalysts as they changed to new forms after dye treatments. A few oxide- and nitride-based materials were reported as highly effective wet air oxidation catalysts of MO degradation, at high MO concentrations (>900 ppm) [2–4 in SI] On the other hand, various metal oxide materials and their composites were applied as photocatalysts in the removal of MO dye from water, being effective in the low concentration range of dye (typically, 20 ppm or lower) [15–19 in SI]. A comparison of various materials used to degrade aqueous MO dye is given in Table S1. To summarize, the Cu-based HDS for the dye removal gave a higher performance than various photocatalysts as they could effectively remove MO from the highly concentrated aqueous solution (500 ppm). Using HDS materials is one of the promising options for pretreatment of highly polluted wastewater.

Among the above metal oxides used to produce Cu-based HDS, CaO can be derived from renewable resources, providing a sustainable alternative to traditional materials from mining. Hence, with the purpose of recycling eggshell waste for high-value purposes, this work focuses on the preparation of biobased CaO from waste eggshells (duck and quail eggshells) at varying calcination temperatures, and the calcined shells were subsequently applied as precursors for the synthesis of CuCa HDS materials via a hydrothermal metal oxide-assisted treatment. Furthermore, the activity of CuCa HDS materials in the removal of MO from aqueous solutions was examined, while the MO removal rates and MO removal efficiencies were compared among the eggshell-derived HDS produced in this work and those in the literature (Table S1). The dye removal processes are discussed based on the structural stability of HDS materials and radical probing techniques. This research involves exploring the optimum eggshell processing temperatures and examining any variation in the activity of CuCa HDS produced from calcined eggshells from different sources. Additionally, the research aims to analyze the effects of different calcination durations on the performance of CuCa HDS in MO removal. The findings will contribute to understanding the potential of using eggshells as a sustainable and cost-effective raw material in catalysis by using MO removal as a representative application.

## 2 Materials and Methods

### 2.1 Materials and Chemicals

Copper nitrate trihydrate (Cu(NO_3_)_2_·3H_2_O) was purchased from Univar, and the MO (C_14_H_14_N_3_NaO_3_S) was purchased from Fischer Scientific. All chemical reagents in this work were analytical grade and used without further purification. Deionized (DI) water was used throughout all of the experiments.

Waste duck eggshells (DES) and quail eggshells (QES) were collected from canteens at Mahidol University, Thailand. These waste eggshells were washed with detergent and tap water to remove dirt and impurities, then dried at 100 °C for 24 h in an oven. The washed eggshells were ground up and sieved to pass through 400 mesh. After that, the fine powder was calcined in a muffle furnace at various temperatures (650, 750, 850, 900, and 950 °C) for 4 h with a heating rate of 10 °C/min. The calcined products (biocalcium source) were kept in a desiccator for future use, and they were labeled based on eggshell source and calcination temperatures; for example, DES_750 refers to the calcined product of DES powder at 750 °C.

### 2.2 Preparation of CuCa HDS

Copper–calcium hydroxy double salt (CuCa HDS) samples were obtained from suspending 1 g of the calcined eggshell products mentioned above in an aqueous copper nitrate solution at the controlled Cu/Ca molar ratio of 4:1 (with the assumption that the major product in calcined eggshells is CaO). The suspensions were sonicated for 30 min in an ultrasonic bath and later transferred into a Teflon-lined stainless-steel autoclave, followed by hydrothermal treatment at 100 °C for 30 min. After that, the reaction mixture was taken out and placed at room temperature to cool down. Precipitates from each suspension were collected by vacuum filtration washing with deionized water, then dried at 65 °C for 24 h, and kept in a desiccator. Preliminary results confirmed the presence of copper hydroxide nitrate in all precipitates. The obtained samples, from the metal oxide-assisted synthesis, are denoted as CuCa HDS, with additional sample codes based on the eggshell precursor and calcination temperature. For example, CuCa HDS_DES_750 was CuCa HDS derived from calcined DES at 750 °C.

### 2.3 Sample Characterizations

The thermal properties of the waste eggshell (DES and QES) powder were analyzed by a thermogravimetric analyzer (PerkinElmer TGA4000) under a gas flow of air zero. Around 10 mg of the eggshell powder was loaded in the TGA analyzer, and the temperature was varied from 30 °C up to 950 °C with a heating rate of 20 °C per min. The specific surface area of the DES and QES powder and their calcined products was analyzed using a MicroActive (ASAP 2460) based on nitrogen adsorption at 350 °C. Note that, the CuCa HDS materials decomposed at around 200 °C, transforming to CuO (as shown in Figure S1). Thus, it was not suitable to measure the surface area of HDS materials by BET analyses, as the degassing and measurement temperatures were higher than 200 °C.

Powdered X-ray diffraction (PXRD) measurement was carried out on a Bruker AXS model D8 Advance diffractometer (40 kV and 35 mA) with CuKα radiation (λ = 1.5406 Å) to characterize the crystal structure of eggshell precursors (QES and DES fine powder), bio-CaO products, and as-synthesized CuCa HDS materials, The XRD patterns of all samples were recorded in 2θ from 10 to 60° with a step of 0.035°/min, and the diffraction patterns were compared to PXRD patterns in the Crystallography Open Database. Attenuated total reflectance-Fourier transform infrared (ATR-FTIR), carried out on a PerkinElmer, was used to identify functional groups in the samples. The IR spectra of the samples were recorded in the range of 400–4000 cm^–1^. The elemental compositions and morphologies of the samples were analyzed by SEM–EDX analysis (FE-SEM HITACHI SU8010). All samples were pretreated by coating them with platinum/palladium for 90 s.

Electron spin resonance spectra were recorded by using 5,5-dimethyl-1-pyrroline *N*-oxide (DMPO) as a spin trapping reagent. Two milligrams of CuCa_HDS were mixed for 2 min with 1 mL of 500 ppm of MO and 1 mL of 50 mM DMPO. After that, the mixture was filtrated to be measured by electron spin resonance (ESR) spectroscopy (Bruker ELEXSYS, ER083CS) following these parameters; X-band standard frequency = 9.846 GHz, modulation amplitude = 1 G, modulation frequency = 100 kHz, and microwave power = 20 mW.

### 2.4 MO Removal

The removal of MO from water was performed in a lid-open test tube, containing 10 mL of 500 ppm of MO(aq) and 20 mg of CuCa HDS material (loading: 2 g/L), magnetic stirring at 1000 rpm under ambient conditions. After the reaction at different times, the treated MO solution was filtered through a syringe filter (nylon, pore diameter 0.45 μm). Following previous studies,^19,20^ the absorbance of treated MO solutions was measured on a UV–vis spectrophotometer (GENESYS 10s UV–vis spectrophotometer). The concentration of the treated MO solution was calculated from the absorbance value at 464 nm, following Beer–Lambert’s law equation. The MO removal efficiency was calculated based on eq 1
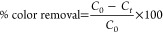
1where *C*_0_ is the initial concentration of MO and *C_t_* is the concentration of MO at reaction time *t* min.

The chemical oxygen demand (COD) removal efficiencies upon MO treatments were assessed by a standard closed reflux/colorimetric method, followed by previous works.^19,20^ The COD removal efficiency is defined as shown in eq 2
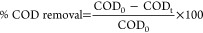
2where COD_0_ is the initial chemical oxygen demand of MO (500 ppm) and COD_*t*_ is the chemical oxygen demand of dye solutions at “*t*” min reaction time.

The stability of the CuCa HDS test was performed by adding the CuCa HDS material to a 500 ppm MO solution with a loading of 2 g/L in a test tube, under magnetic stirring (1000 rpm) for 5 min, centrifuging for 10 min, and syringe filtration to separate the treated solution and the spent CuCa HDS. The treated solution was subjected to the UV–vis spectrophotometer to calculate color removal efficiency. The spent CuCa HDS was subsequently added to a fresh MO solution for the next run, followed by the same process until the color removal efficiency became lower than 50%. Then, the spent CuCa HDS, at that stage, had a low activity in MO removal. An acid wash method was utilized to regenerate the inactive CuCa HDS (after the fourth cycle). The spent, inactive CuCa HDS was suspended in a 5 mM HCl(aq) solution and stirred for 30 min before being rinsed with water. Then, the acid-washed HDS was applied in the fifth run of 500 ppm MO(aq) treatments, while the color removal efficiencies were measured.

## 3 Results and Discussion

### 3.1 Sample Characterizations

**Figure 1:**
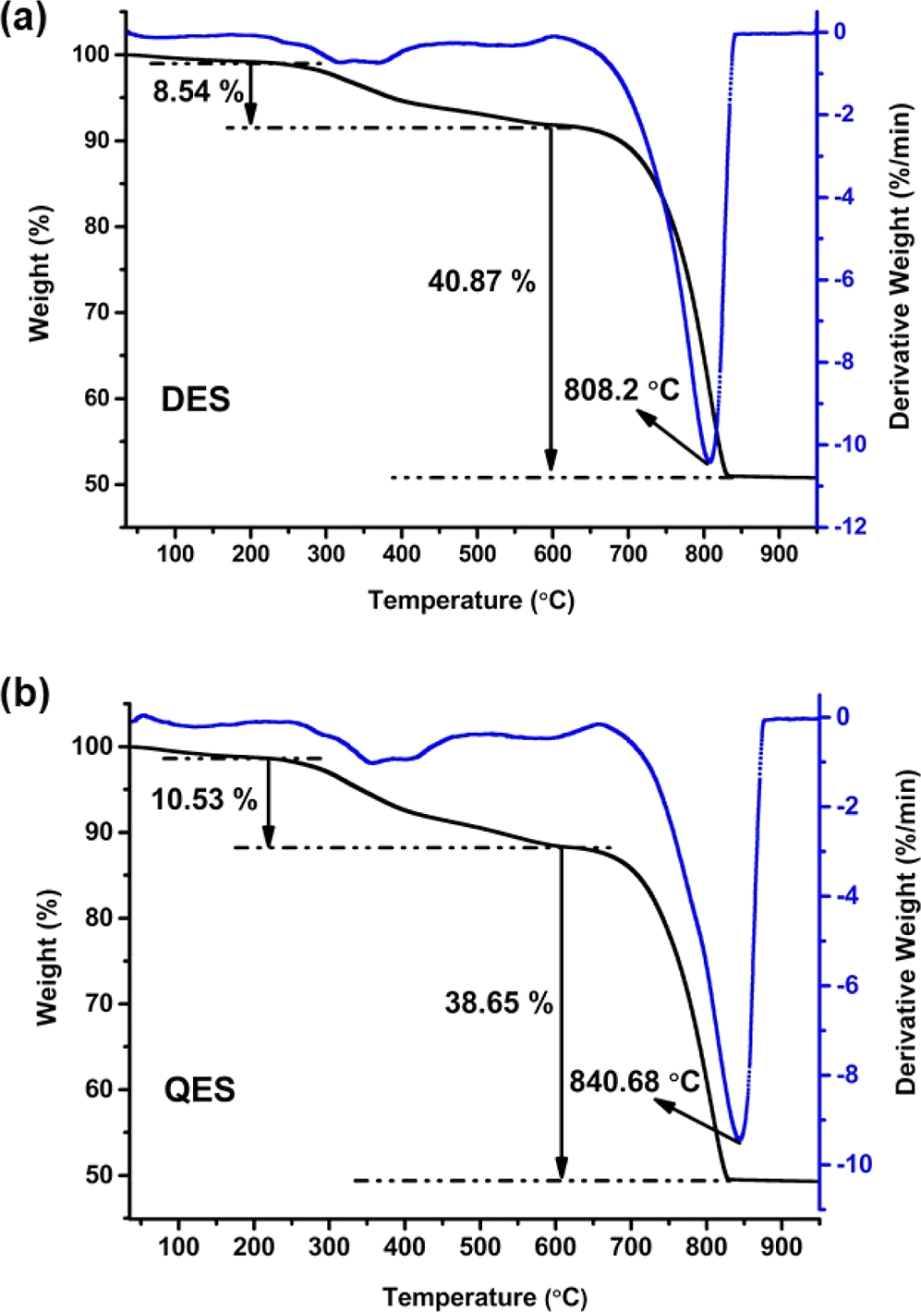
TGA profiles of (a) DES and (b) QES.

Eggshells are widely known as one of the calcium carbonate (CaCO_3_) sources, and thermal treatments lead to the decomposition of CaCO_3_ into CaO or quicklime. Dried eggshell powdered samples derived from DES and QES were analyzed by thermogravimetric analysis to evaluate their thermal stability. The results in Figure 1 illustrate that the eggshells decomposed, giving two main weight loss steps. First, a weight loss of 8.54 and 10.53 wt % was observed for DES and QES, respectively, between the temperature range of 30–650 °C attributed to the removal of water and the decomposition of organic matter. Second, the weight loss at the temperature between 650 and 850 °C, 40.87 wt % and 38.65 wt % weight loss, for DES and QES is expected to be the decomposition of CaCO_3_ and the release of CO_2_.^21,22^

Further heating from 850 to 950 °C brought negligible change to both samples. Based on the amount of CO_2_ gas released in the second step, it is possible to evaluate the CaCO_3_ and Ca content in each eggshell sample. Consequently, the DESs contain 92.89 wt % of CaCO_3_ or 37.15 wt % of Ca, while the QESs have 87.84 wt % CaCO_3_ or 35.13 wt % Ca. The derivative TG plots (blue line in Figure 1) suggested a relatively high energy consumption for the CaCO_3_ to CaO conversion in the QESs. Such conversion was completed at around 841 °C for QESs, which is 32 °C higher than that for DESs. However, in other studies, temperatures at 924 °C or above were required to completely convert CaCO_3_ in an eggshell sample to CaO.^5,6^ Calcium oxide from eggshell waste has been classified as similar to calcium oxide from limestone, and it can be a CaO source for many industries.^6^ Therefore, the conversion of DES to lime may have advantages over QESs as the requirement for energy input is lower.

**Figure 2:**
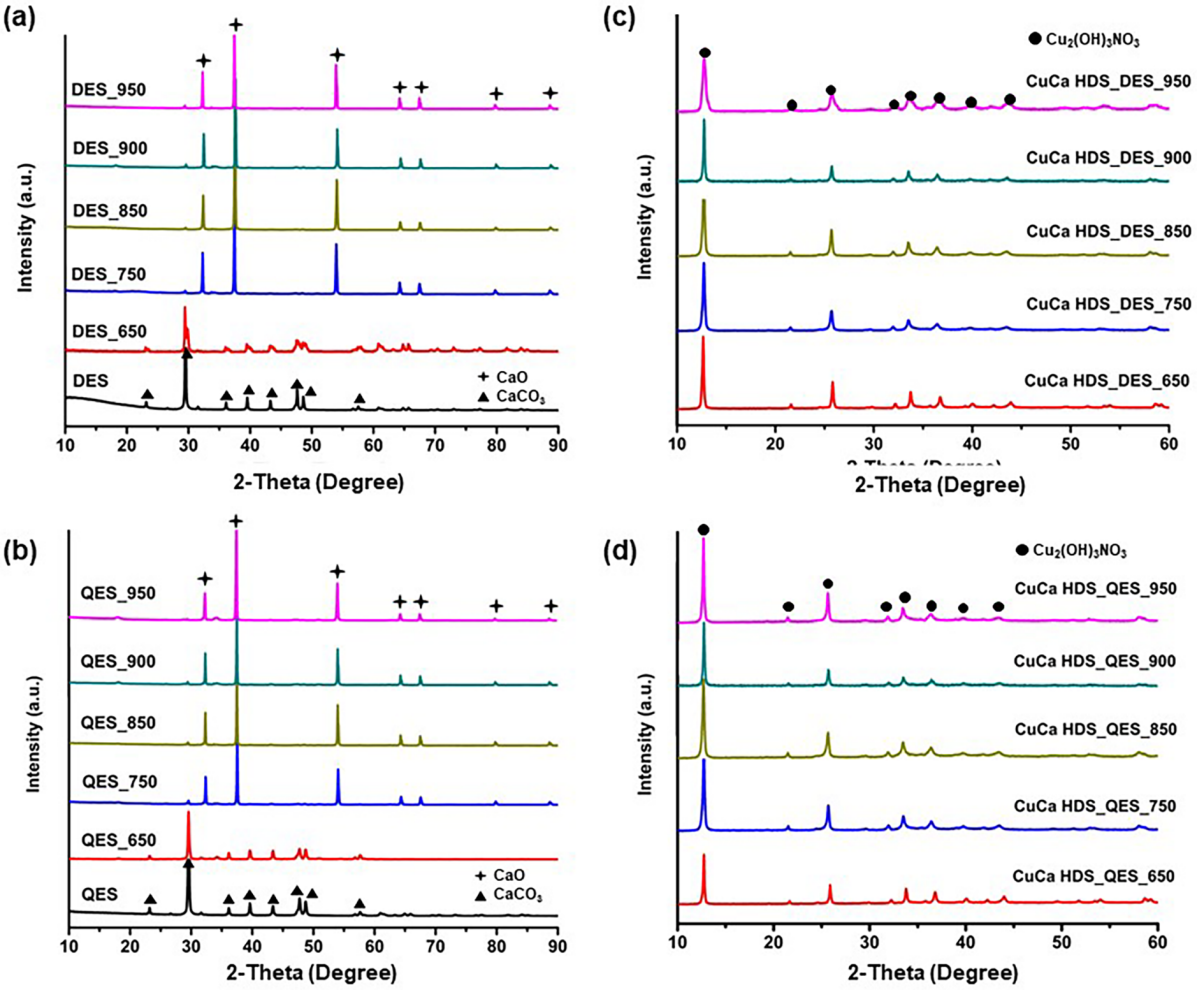
PXRD patterns of (a) DES, (b) QES series, and (c,d) as-synthesized CuCa HDS materials.

In Figure 2a, DES gave a powder XRD profile having peaks at 2θ = 23.2, 29.5, 36.1, 39.5, 43.3, 47.6, and 48.6°, which were the characteristic peaks of calcite-CaCO_3_ (Crystallography Open Database standard no. 1010962), being similar to the reported results by Risso et al.^21,23^ By being calcined at 750 °C, the XRD peaks corresponding to CaO were found at 2θ = 32.3, 37.4, 53.9, 64.3, 67.4, 79.8, and 88.6°, matching the COD standard no. 1011095. With a further increase in the calcination temperature from 850 to 950 °C, the characteristic peaks of CaO for calcined DES were evident and insignificantly different from each other. Agreed well with the TGA results, the weight loss of DES at temperatures above 833.32 °C was negligible. The results from Figure 2a,b suggested that crystalline CaO derived from both eggshells started to form at 750 °C. The observed XRD peak at 29.5° suggests CaCO_3_ trace is still present even at 950 °C. Subsequently, the calcined eggshell powdered samples were employed to prepare CuCa HDS.

Figure 2c,d confirms the successful formation of CuCa HDS samples derived from the calcined eggshells. It showed that all samples exhibited typical characteristic peaks of copper hydroxide nitrate or Cu_2_(OH)_3_NO_3_ (COD Standard no. 9012715), with sharp peaks at around 2θ = 12.9 and 25.9°, which indexed to (001) and (002) basal planes, and the value of interlayer distances was *d*_(001)_ = 6.9 Å, *d*_(002)_ = 3.4 Å.^24−26^ Although the calcined eggshell materials contain CaCO_3_ and CaO, the formation of a CuCa HDS single phase from all eggshell precursors was successful. In addition, no diffraction peaks associated with CuO, Cu_2_O, Cu(OH)_2_, or CaCO_3_ were detected. Note that when DESs heated to 950 °C (DES_950) were used as a raw material for HDS, the resulting HDS materials were less crystalline than those made from the eggshells calcined at lower temperatures. Figure S1 depicts DES_950 particles with a high degree of agglomeration. Because HDS was formed via exchange reactions between CaO and Cu(NO_3_)_2_, the lower crystallinity of CuCa HDS DES_950 may be due to the slower dissolution rate of highly agglomerated DES 950.

**Figure 3:**
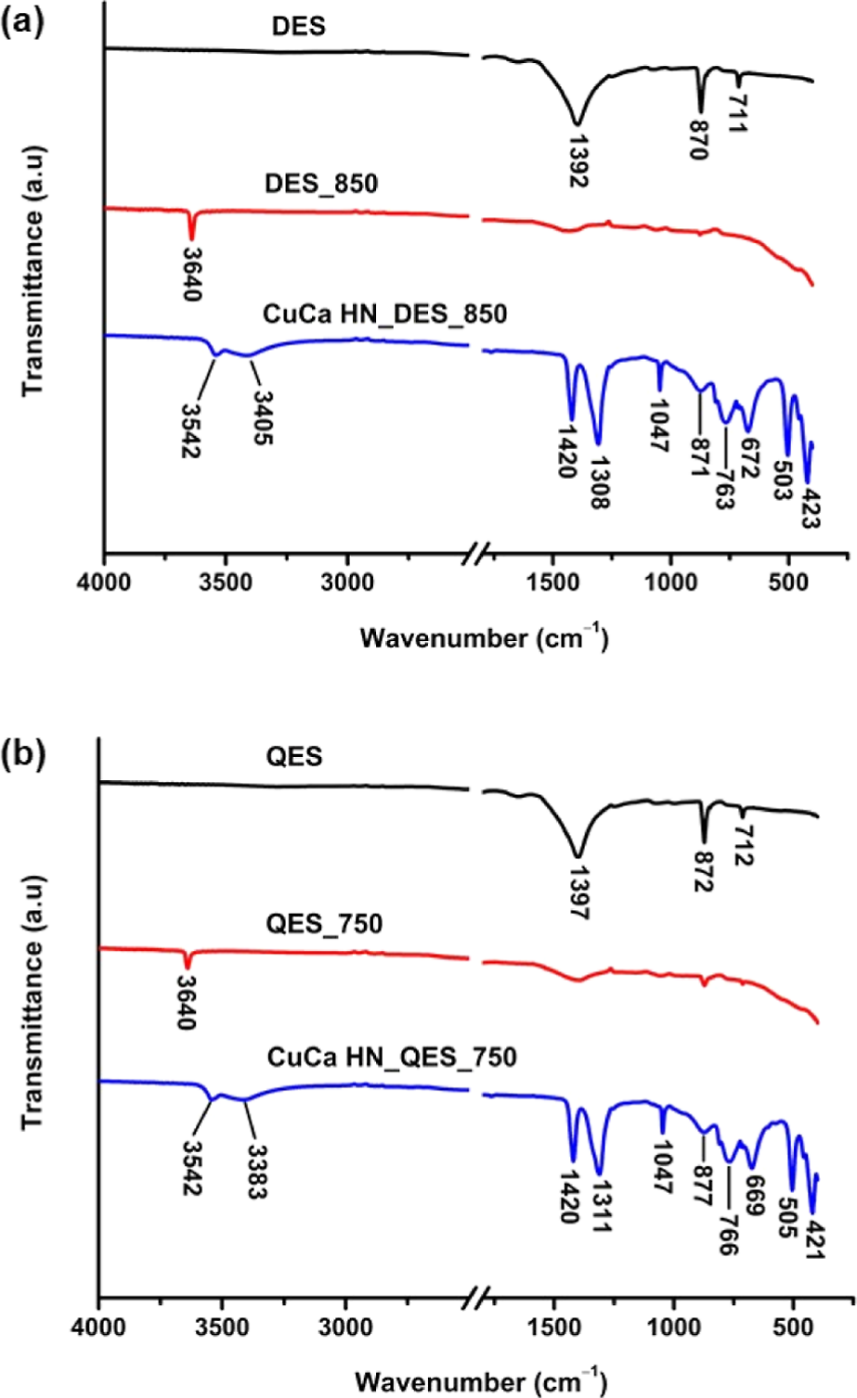
FTIR spectra of (a) DES series and their corresponding CuCa HDS materials and (b) QES series and their corresponding CuCa HDS materials.

FTIR analysis was performed for the eggshell and CuCa HDS samples to determine their chemical compositions. In Figure 3a,b, the FTIR spectra suggest that the DES and QES series have similar functional groups and chemical entities, according to the evident absorption bands at around 1392–1397, 870–872, and 711–712 cm^–1^.

For uncalcined DES, the absorption band centering around 1392 cm^–1^ was associated with the asymmetric stretching of carbonate groups; the two sharp peaks at 870 and 711 cm^–1^ relate to the out-plane and in-plane bend vibration modes of CO_3_^2–^ groups, respectively. After calcination, the absorption band of CO_3_^2–^ obtained in calcined DES and QES products became weaker due to the loss of carbonate. In addition, the absorption peak at 3640 cm^–1^ observed in each calcined sample is possibly related to the O–H stretching vibration of the Ca(OH)_2_ formed from moisture absorbed on the surface of the calcined eggshell powder.^6,27,28^ Furthermore, the formation of Cu_2_(OH)_3_NO_3_ can also be confirmed by the IR spectra (Figure 3a,b) of CuCa HDS derived from the calcined eggshells. Figure 3a depicts that adsorption bands centered at around 3542 and 3405 cm^–1^ were indexed to stretching of the O–H from molecular water in the interlamellar space and to hydrogen-bonded O–H groups in the layered structure. The IR bands at 1420, 1308, and 1047 cm^–1^ are associated with the vibrational modes of the NO_3_^–^ group. The hydrogen bonding frequencies related to the bending vibrations of Cu–O–H are observed at IR bands of 871, 763, and 672 cm^–1^. The IR bands at 503 and 423 cm^–1^ might be associated with metal–oxygen bonds. The IR spectra of CuCa HDS derived from the calcined QES are shown in Figure 3b, which showed similar adsorption bands to those of CuCa HDS derived from the calcined DES.^19,20,29−31^ Overlapping IR peaks made it difficult to identify any carbonate ions in the CuCa HDS materials.

The chemical compositions and specific surface areas of DES, QES, and their calcined products are given in Table 1. The Ca content in the uncalcined eggshells from EDX is different from TGA data since EDX detects the elemental composition at the surface. However, the similar Ca content in the CuCa HDS produced from each eggshell source is less dependent on the calcination temperatures.

**Table 1:** Specific Surface Area and Elemental Composition of the Uncalcined and Calcined Eggshell Surface Analyzed by EDX^a^

sample	Ca (% w/w)TGA	specific surface area (m^2^/g)
DES	33.00	6.2
DES_750	39.87	3.4
DES_850	42.51	19.9
DES_900	41.96	19.3
DES_950	46.51	8.4
QES	50.71	4.4
QES_750	43.90	7.5
QES_850	42.19	10.4
QES_900	46.28	13.7
QES_950	39.12	1.1

a The standard deviation error for Ca is within 4%.

In addition, the specific surface area of uncalcined eggshells is quite low, less than 1 × 10 m^2^/g. The heat treatments led to gas release and a slight increase in the specific surface area of calcined eggshells, as shown in Table 1. The largest surface area of 19.93 m^2^/g was detected from the DES calcined at 850 °C. The low specific surface area of the eggshell calcined at 950 °C might be caused by the sintering effect from prolonged heating at a higher temperature.^32,33^ Moreover, EDX analysis was employed to determine the elemental composition at the CuCa HDS surface, as shown in Table 2. Notably, similar IR and XRD data were obtained from the CuCa HDS produced from different eggshell sources, and the CuCa HDS prepared from the calcined eggshell shows quite similar Cu content and Ca trace.

**Table 2:** Elemental Composition of the CuCa HDS Surface Analyzed by EDX^a^

sample	Cu	Ca	N
CuCa HDS_650	28.42	0.03	9.05
CuCa HDS_750	46.49	0.15	7.26
CuCa HDS_850	45.42	0.11	8.36
CuCa HDS_900	45.66	0.28	6.67
CuCa HDS_950	51.12	0.24	6.73
CuCa HDS_1050	51.73	0.20	4.91

a The standard deviation error for Cu is within 4.4%, for Ca is within 0.42%, and for N is within 2.5%.

There is no evident correlation between the chemical composition and the BET surface area of eggshell precursors calcined at different temperatures. Noticeably, a low Cu content was observed for CuCa HDS_DES_650 and CuCa HDS_QES_650 in comparison to other CuCa HDS samples. This may be the major CaCO_3_ phase in the eggshells calcined at 650 °C. The previous work^20^ and Li and Zhang et al.^34^ reported that the higher copper contents in LDH promoted more effective MO (aq) decolorization.

**Figure 4:**
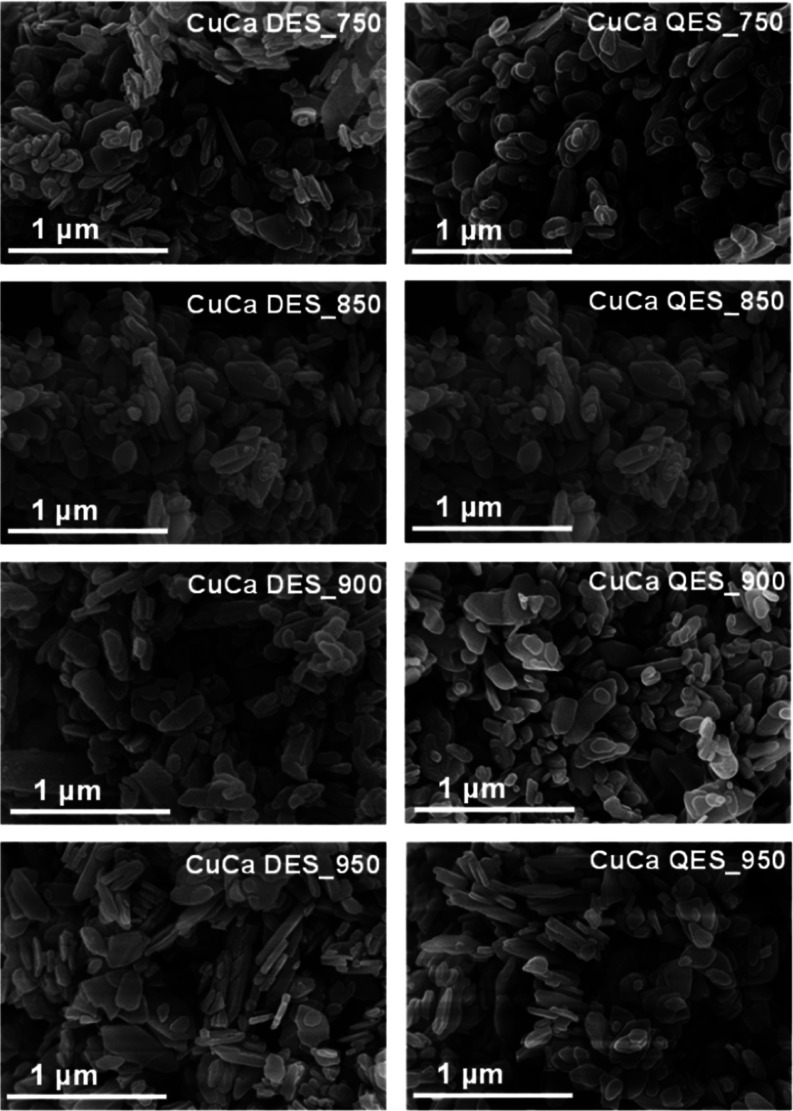
Morphology of CuCa HDS derived from calcined DES and QES.

The morphologies of uncalcined DES, QES, and their calcined products were characterized and are shown in Figure S3. The uncalcined eggshells exhibit a rough surface with large, irregular-shaped particles. After calcination, small irregular-shaped crystal forms were found. In addition, at calcination temperatures of 750 and 850 °C of QES and DES, respectively, they had irregular sizes of rod particles. All calcined eggshells showed a crack and rough surface, perhaps due to the decomposition of CaCO_3_ and the emission of CO_2_, leading to the smaller particle sizes.^35^ The SEM images of CuCa HDS derived from eggshells are shown in Figure 4, showing irregular sizes of plate-like particles with a smooth surface. The morphology and crystallinity of CuCa HDS materials are similar, independent of the Ca sources.

### 3.2 Decolorization of MO

**Figure 5:**
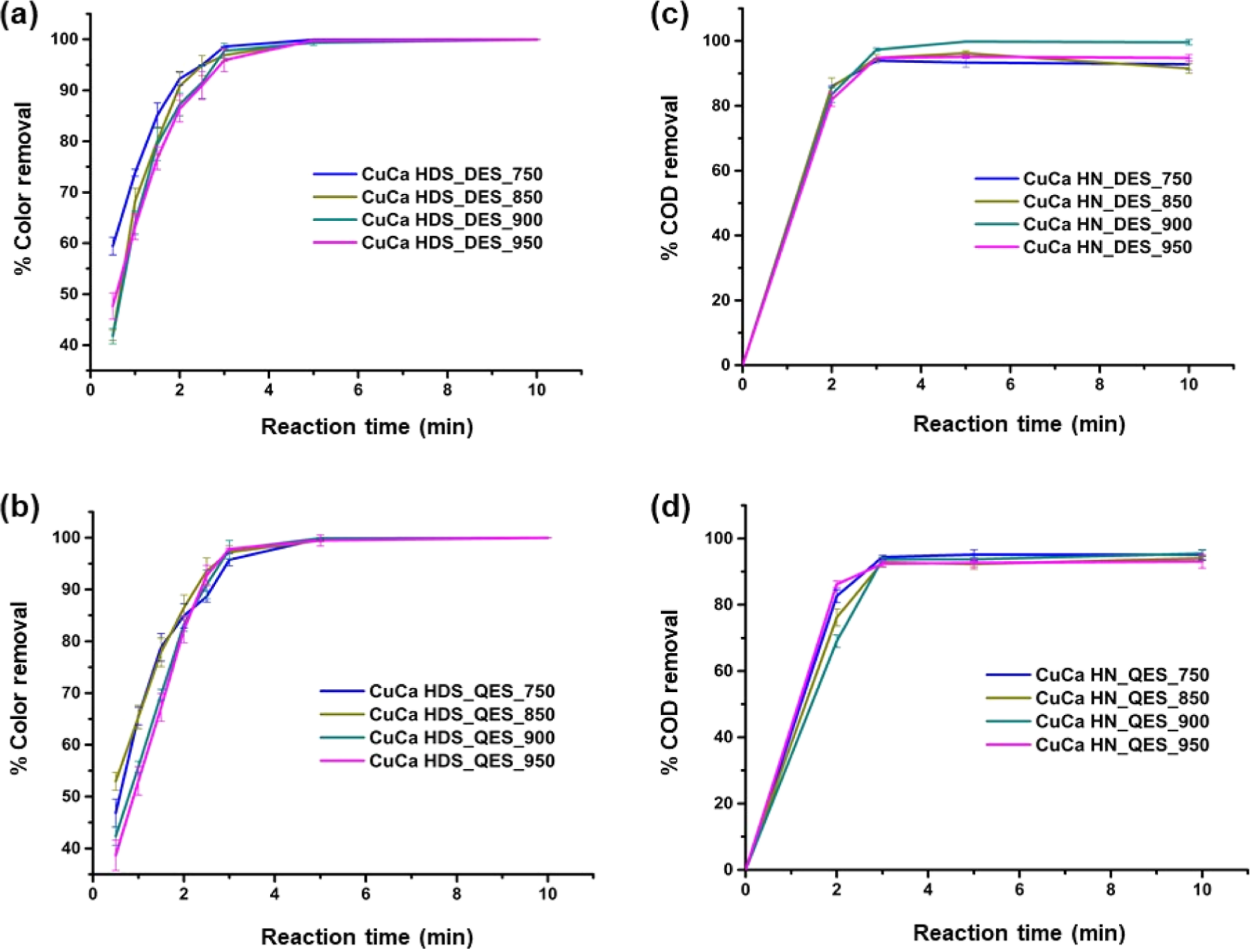
Color removal efficiencies versus treatment time of MO(aq) with the CuCa HDS from calcined DES (a) and QES (b) and % COD removal efficiency with time using the CuCa HDS derived from calcined DES (c) and QES (d). [MO] = 500 ppm, loading: 2 g/L, under ambient conditions.

The activity of CuCa HDS materials in the decolorization of MO solution was examined over a function of time under ambient conditions, as shown in Figure 5a,b. Rapid color removal gave color removal efficiencies of more than 83 and 99% just after 2 and 5 min, respectively.

The reaction rate of decolorization was evaluated and matched with the pseudo-first-order kinetic model (eq 3 and Table 3).
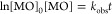
3

**Table 3:** Pseudo-First-Order Rate Constant (*k*_obs_) for MO Decolorization over Various CuCa HDSs Are Given with the Fitting *R*^2^^a^

sample	*k*_obs_ (min^–1^)	*R*^2^	color removal efficiency after 2 min
CuCa HDS_DES_650	0.55	0.9658	75.46 ± 1.42
CuCa HDS_DES_750	1.11	0.9923	92.28 ± 1.30
CuCa HDS_DES_850	1.20	0.9900	90.89 ± 2.85
CuCa HDS_DES_900	1.02	0.9991	87.26 ± 2.23
CuCa HDS_DES_950	0.90	0.9913	86.43 ± 2.62
CuCa HDS_QES_650	0.37	0.9933	63.73 ± 1.96
CuCa HDS_QES_750	0.85	0.9951	84.92 ± 2.30
CuCa HDS_QES_850	0.84	0.9896	86.49 ± 2.45
CuCa HDS_QES_900	0.82	0.9676	83.29 ± 1.34
CuCa HDS_QES_950	0.82	0.9632	82.32 ± 2.59

a The color removal efficiencies of 80% and above were achievable when eggshells calcined at temperatures of 750 °C and above were used as the precursor.

Given in eq 3, [MO] is the concentration of MO at time *t*, while [MO]_0_ is the initial concentration of MO (500 ppm in this study), and *k*_obs_ is the pseudo-first-order rate constant.

The pseudo-first-order kinetic model, an assumption that allows the rate equation to be approximated to a simple first-order rate equation where the reaction rate is directly proportional to the concentration of the MO (eq 3), has been widely addressed in several investigations (Table S1). From the results, eggshells calcined at 650 °C gave the CuCa HDS that was less active in MO decolorization compared to other calcined eggshell samples. This is due to the low Cu content (Table 2) in CuCa HDS_*x*ES_650 (*x* = D or Q) and the high CaCO_3_ content in the calcined eggshells (suggested by TGA data). Similar to a previous work,^20^ the Cu-based hydroxide nitrate with high Cu content are more active materials for dye decolorization. Based on the reaction rate data, the calcined DES gave the more active CuCa HDS for MO decolorization than the calcined QES. The small variation of the MO decolorization rates among the calcined eggshells of the same source was observed, suggesting that the insignificant calcination temperature (750 °C and above) was dependent on the activity of CuCa HDS. The COD analysis determines the quantity of oxygen required to oxidize the organic matter in water. Figure 5c,d shows that highly effective COD removal (>90%) was achieved after 5 min of MO removal in the presence of eggshell-derived CuCa HDS materials. Thus, such highly polluted water (COD = 561 mg O_2_/L for 500 ppm MO) can be effectively purified by using eggshell-derived CuCa HDS materials.

### 3.3 The CuCa HDS Structural Stability

**Figure 6:**
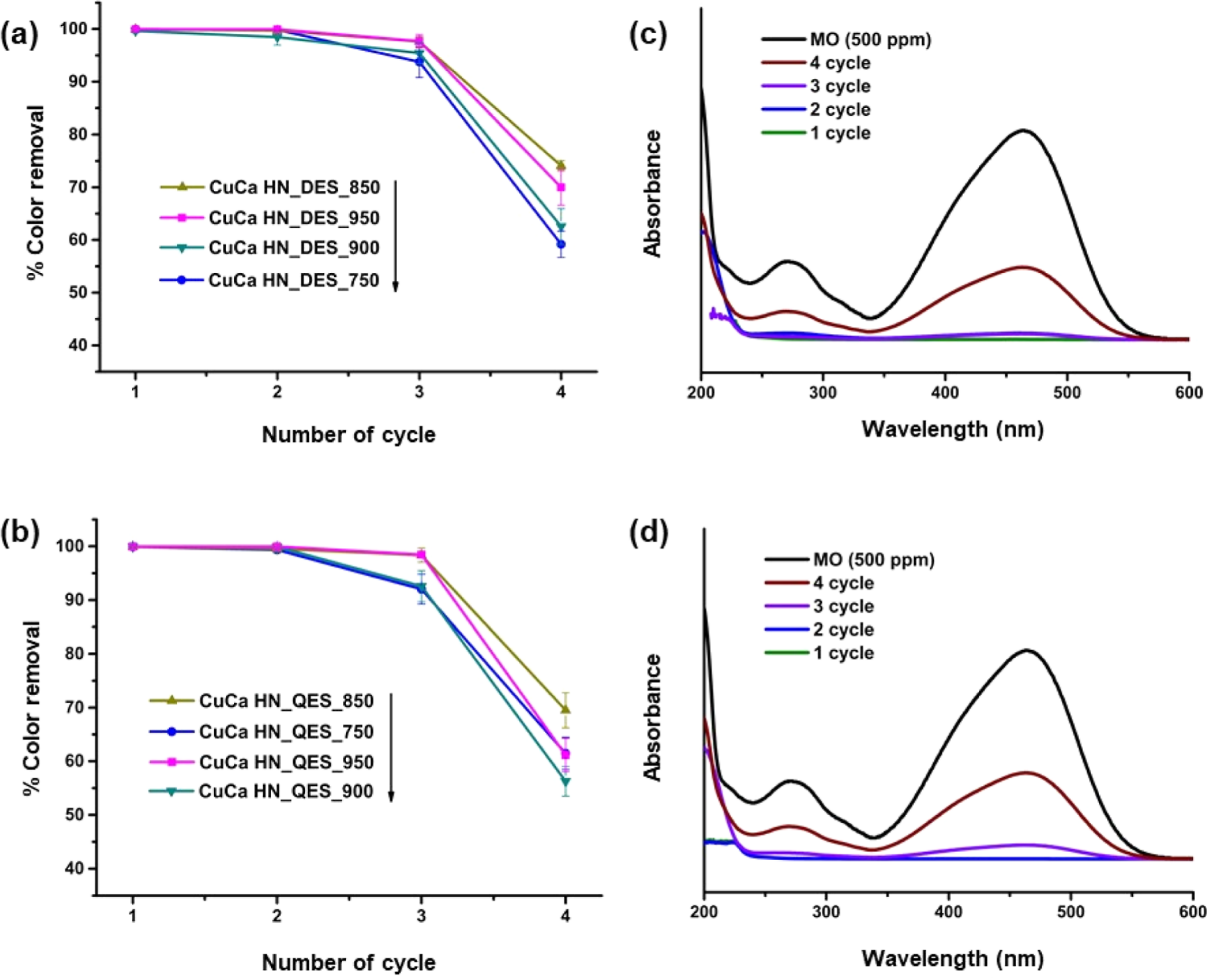
Reusability profiles for derived CuCa HDS materials from calcined DES (a) and QES (b) in color removal efficiency of MO solution for 5 min, (c,d) UV–vis absorption spectrum of fresh MO (500 ppm), and those of oxidized MO after each cycle reaction within 5 min using (c) CuCa HDS_DES_900 and (d) CuCa HDS_QES_900. [MO] = 500 ppm, loading: 2 g/L, under ambient conditions.

The stability of eggshell-derived CuCa HDS was studied by examining the MO decolorization activity in subsequent cycles. The spent CuCa HDS was suspended in a 500 ppm MO(aq) in the next runs, and the MO color removal efficiencies over the spent CuCa HDS are reported in Figure 6a,b. It was found that the spent CuCa HDS gave high MO removal efficiencies in the first two cycles and slightly lower ones in the third one.

However, even after three cycles, more than 90% of MO removal efficiencies were achievable. Thus, the CuCa HDS materials can be reused continuously for three cycles without washing or regeneration requirements. When further reusing the CuCa HDS in the fourth run, the color removal efficiency dropped significantly, *ca*. 55–75% of the MO removal efficiencies. In the fourth cycle, the eggshells calcined at 850 °C gave the most active CuCa HDS material in MO removal (Figure 6a,b). The loss of HDS material during the separation of the supernatant and the buildup of MO and other byproducts on the surface of the HDS, which blocks the active sites, could be causing the decreased MO removal rates. Figure 6c,d depicts the UV–vis spectra of the treated MO solution from each cycle of the CuCa HDS treatments by comparison with fresh MO solution (500 ppm), choosing CuCa HDS_DES_900 and CuCa HDS_QES_900 as their reaction rates stay at an average level between each series of eggshell-derived materials. The absorption spectrum of the untreated MO(aq) showed two main peaks at 465 and 272 nm, which are associated with azo linkage and the benzene ring, respectively.^36^ After the first cycle of 5 min CuCa HDS treatment, the intensity of both peaks became much lower, indicating the cleavage of azo bonds and the benzene ring in the MO molecule, resulting in decolorization. There was no spectral shift corresponding to a possible complexation between dye molecules and metal cations that was observable. According to the decreased color removal efficiency of MO in the fourth run, the spent material collected was found to have a color change from light blue to orange, which suggested MO adsorption on the CuCa HDS surface.

**Figure 7:**
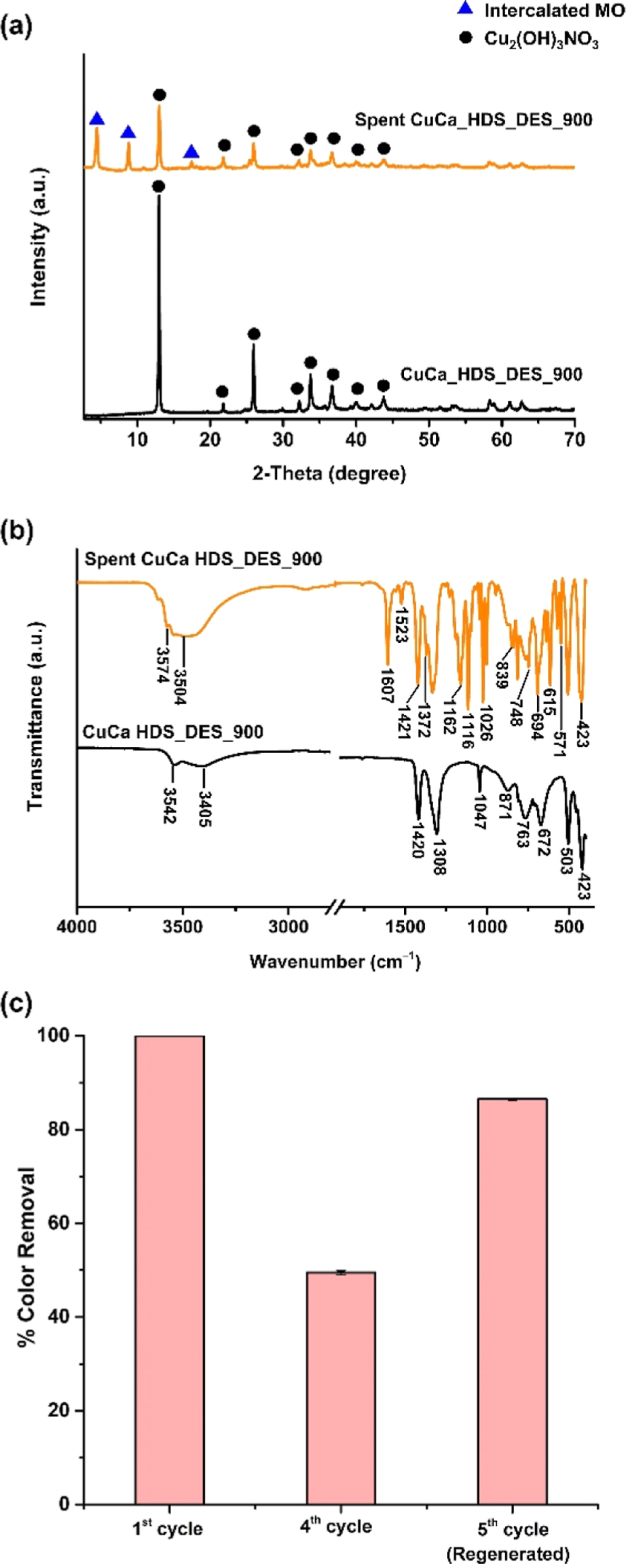
(a) PXRD pattern of spent CuCa_HDS_DES_900 after treatment with MO for 5 min compared with as-prepared CuCa_HDS_DES_900. (b) FTIR spectra of the as-prepared and spent CuCa_HDS_DES_900. [MO] = 500 ppm, loading: 2 g/L, under ambient conditions. (c) Color removal efficiencies from the 5 min MO treatments with CuCa_HDS_DES_850, from the first and the fourth cycle. After the fourth cycle, the HDS material was acid-washed.

In addition, the structure of HDS changed because of the intercalation reactions between MO dye molecules and the nitrate anions in HDS. As seen in Figure 7a, the exchange between nitrate and MO results in new diffraction peaks at 2θ = 4.4° observed in the spent HDS materials, which corresponds to a new *d*-spacing of 19.6 Å, being much higher than the brucite-like layer thickness of typical HDS materials (4.8 Å).^37^

Such newly observed interlayer spacing was comparable to the MO dimensions, as supported by a previous study.^38^ With a new peak at 8.8 also referring to MO intercalated structure, the diffraction peak at 17.5° referred to MO crystalline adsorption on the surface of HDS, similar to the previous study.^38^ The typical FTIR spectra in the region 4000–500 cm^–1^ of the spent materials of CuCa HDS_DES_900 demonstrated the changes of functional groups after utilization in the third run for MO removal as shown in Figure 7b. Compared with the as-prepared CuCa HDS, the characteristic absorption bands related to Cu_2_(OH)_3_NO_3_ were present for the spent HDS, with some additional absorption peaks. New peaks appearing at 1607 and 1523 cm^–1^ were assigned to the MO aromatic rings. The peak centered at 1421 cm^–1^ was associated with the nitrate group in the HDS, whereas the peak at 1372 cm^–1^ might be attributed to the C–N vibration in MO. The spent HDS material has a new peak appearing at 1162 cm^–1^, possibly associated with C–O groups for alcohol, as suggested by Hua and co-workers.^35^ While absorption peaks at 815 and 694 cm^–1^ are related to the C–H stretching vibration of the benzene rings in MO, the spent sample shows additional peaks at 839, 748, and 615 cm^–1^, which can be attributed to substituted benzene.^35,36^ The additional peak at 571 cm^–1^ found from the spent HDS material corresponded to the C–S stretching vibration of the degraded product with a sulfonated aromatic ring.^36,39^ Thus, the IR results suggested MO azo bond cleavage, benzene ring decyclization, and desulfurization during the CuCa HDS treatments, while MO and MO degradation products attached to the CuCa HDS surface cause HDS deactivation in the MO removal.^40^ The spent HDS material was regenerated by washing with a dilute HCl acid solution, while the MO and MO degradation products were washed off, giving a cleaner HDS surface. Subsequently, the high activity of HDS for effective MO removal was restored to 87% in the fifth run, as shown in Figure 7c.

**Figure 8:**
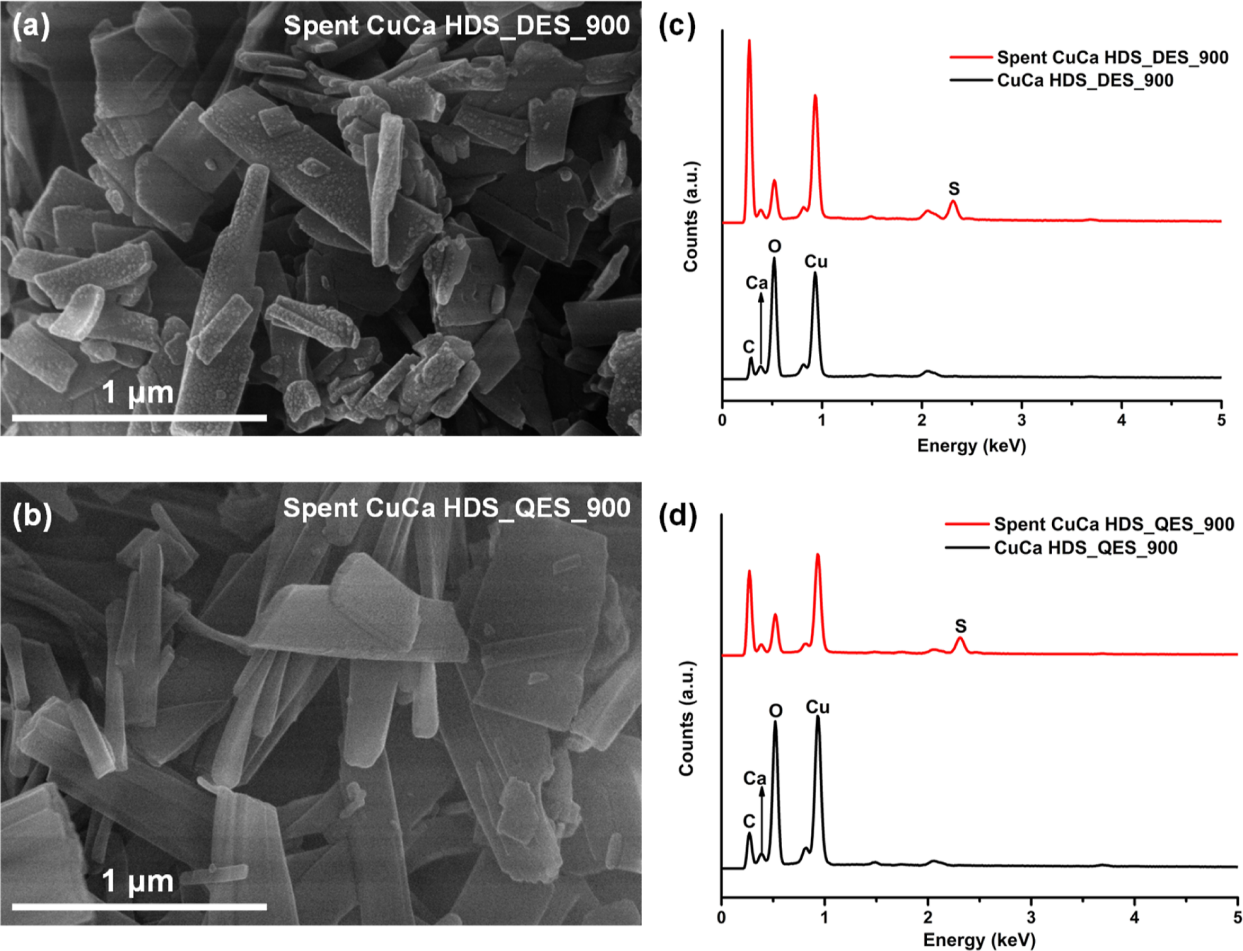
SEM images of the spent (a) CuCa HDS_DES_900 and (b) CuCa HDS_QES_900 and EDX spectra of the fresh and used CuCa HDS materials (c) CuCa HDS_DES_900 and (d) CuCa HDS_QES_900.

Figure 8a,b displays the morphologies of the as-prepared and spent CuCa HDS samples after the third run of the MO oxidation reactions without regeneration. A slight increase in the length of the spent HDS material compared to the as-prepared HDS materials was observed, which could be the result of MO intercalation, as previously discussed (Figure 7a). With the EDX data shown in Figure 8c,d, the detectable sulfur content from spent HDS materials was due to the absorbed MO and MO degradation products on the HDS surface. This work performed the MO removal tests without applying any regeneration processes. To improve the HDS activity in MO removal, regeneration methods can be applied, such as ethanol^41^ or acetone^42^ washing, or a combination of HCl washing followed by calcination,^36^ or a combination of water washing followed by calcination.^43^

As reported previously, several HDS materials served as oxidative catalysts for hydroxyl radical-assisted degradation of organic compounds.^19,20,38^ Hence, ESR experiments were performed to monitor any ^•^OH radicals formed during the CuCa_HDS_DES treatments of MO(aq).

**Figure 9:**
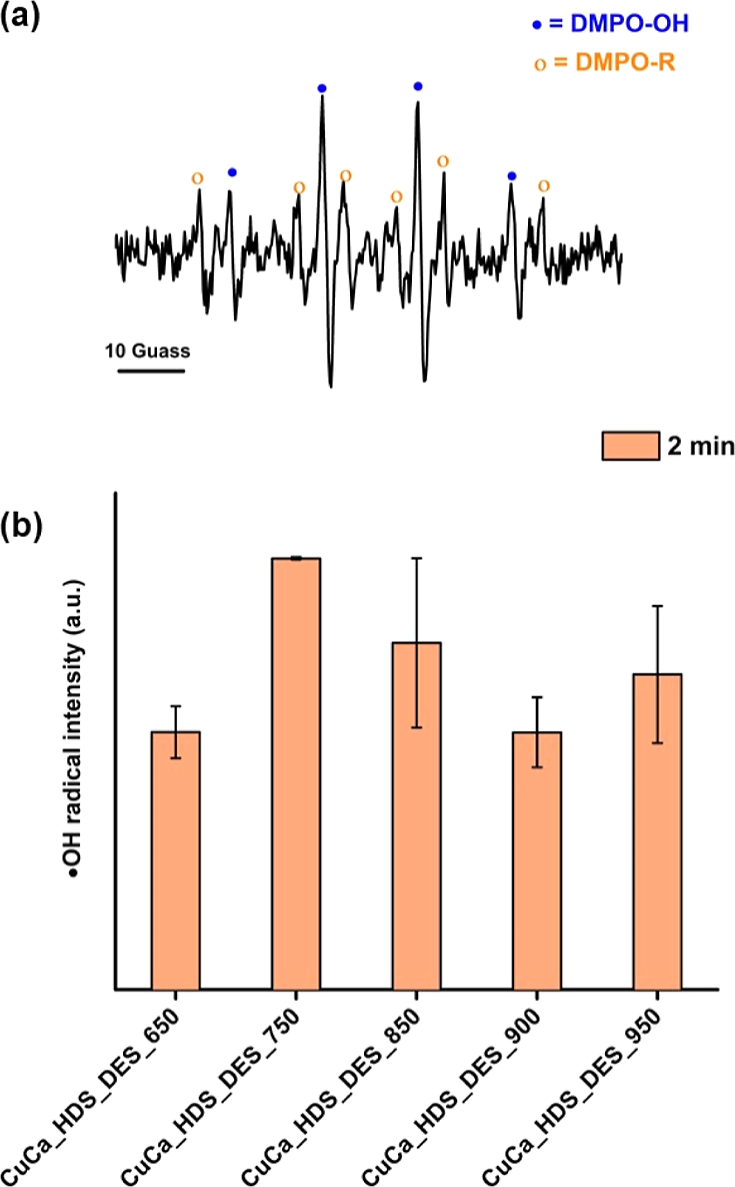
(a) ESR profile of the DMPO adduct in the reaction between CuCa_HDS_DES and MO for 2 min and (b) DMPO–OH adduct intensity.

Figure 9a presents the ESR profile of the DMPO adduct with hydroxyl radicals (DMPO–OH) and carbon-centered radicals (DMPO-R). The hyperfine coupling constant of adducted DMPO–OH (*g* = 2.0057) was *a*_N_ = 15.0 G and *a*_H_ = 14.7 whereas the hyperfine coupling constant of adducted DMPO-R (*g* = 2.0055) was *a*_N_ = 15.6 *a*_H_ = 22.6 G.^44,45^ The amount of ^•^OH radicals (extracted from the intensity of ESR signals corresponding to DMPO–OH) in the treated MO solutions after the 2 min CuCa HDS_DES treatments is reported in Figure 9b. These results confirmed the presence of radical species in the treated MO solutions. Based on the results shown in Figures 7 and 9, the removal of MO from water by CuCa HDS involved adsorption, intercalation, and hydroxyl radical-assisted oxidation. The intercalation of MO molecules within the CuCa HDS structure further contributes to the effective removal process. Nevertheless, the detected amount of hydroxyl radicals could not be used to describe the relative activity of CuCa HDS_DES materials at 2 min treatments (Figure 5a,b) due to the rapid and comparable MO and COD removal rates that were achievable for all HDS materials.

**Figure 10:**
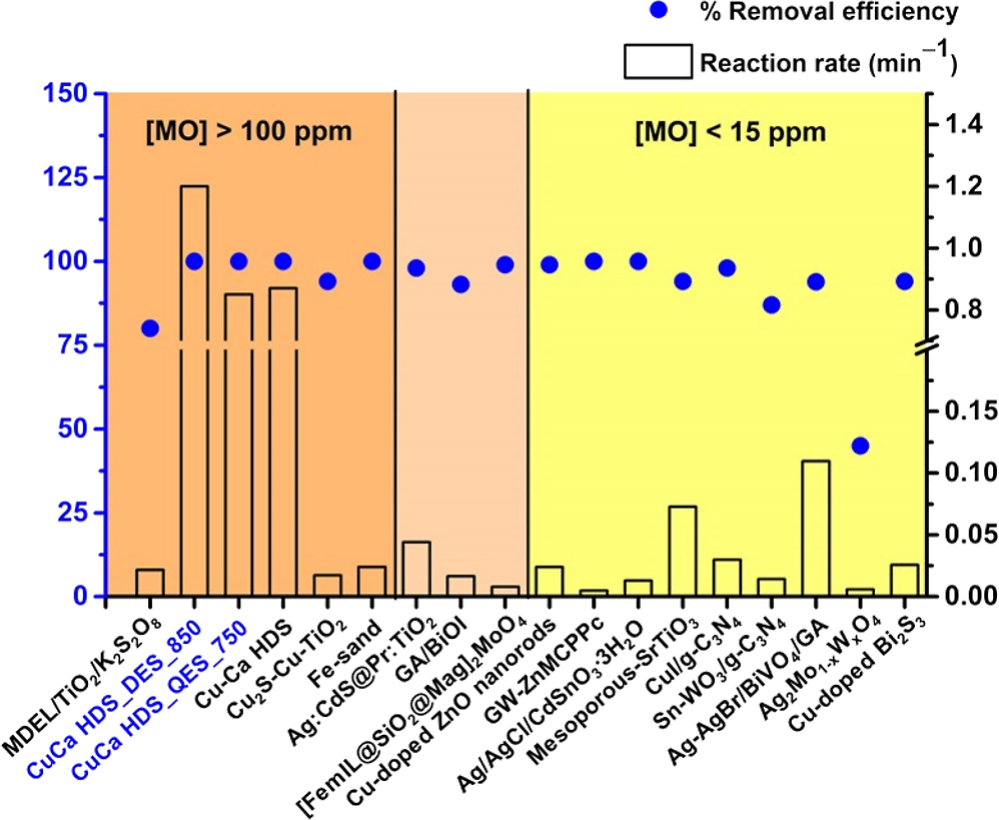
Pseudo-first-order reaction rate (min^–1^) and % removal efficiency of the CuCa HDS materials and other materials used on degradation of MO dye.

Various solid materials have been widely employed to catalyze MO(aq) degradation, and kinetic parameters were reported and given in Table S1. Li^34^ and Tao^46^ utilized layered double hydroxide (LDH) materials as catalysts for the complete removal of MO from water under heating and H_2_O_2_ addition. Other catalysts, such as MnO_2_/CeO_2_,^47^ Au/Fe_3_O_4_,^48^ and CuO/CeO_2_,^49^ have high catalytic activity in MO decolorization within short treatment times; however, they require heating and additional H_2_O_2_ as an oxidant. Furthermore, g-C_3_N_4_/Au^50^ effectively treated MO dye within a short time under ambient conditions, but the gold precursor is expensive. Next, CuO/γ-Al_2_O_3_^35^ has been reported to have good catalytic activity in the CWAO processes for the degradation of the aqueous MO dye at a high concentration. However, the reactions require a high temperature to achieve complete MO removal. Zhang et al.^51^ reported that Cu–Fe–La/γ-Al_2_O_3_ gave high COD removal efficiencies upon their treatment of concentrated MO dye solution via the CWAO method, in which they are reusable for five cycles; however, the process requires high temperature and pressure. On the other hand, Fe_2_O_3_–CeO_2_–TiO_2_/γ-Al_2_O_3_^36^ gave an MO removal efficiency of 98.09% after 150 min, with the requirement of airflow (oxygen acted as an oxidant). From those reports and what was obtained from this work, the pseudo-first-order reaction rate (min^–1^) and % removal efficiency for the degradation of MO dye by CuCa_HDS_DES_850, CuCa_HDS_QES_750, and other materials, are shown in Table S1 and Figure 10 compared with those in previous studies.

The initial MO concentration ranges are included in Figure 10 to further discuss the material’s performance in MO decolorization comparatively. Three ranges of the initial MO concentration include: (i) high concentration: [MO] > 100 ppm, (ii) medium concentration: 100 ppm > [MO] > 15 ppm; and (iii) low concentration: [MO] < 15 ppm. The CuCa_HDS_DES_850 and CuCa_HDS _QES_750 treatments are listed in the high initial concentration of MO(aq) ([MO] > 100 ppm). As reported in Figure 10, the CuCa HDS materials investigated in this work gave high reaction rates and MO removal efficiencies compared with those obtained from other materials. For example, CuCa_HDS_DES_850 and CuCa_HDS_QES_750 gave reaction rates of 1.20 and 0.85 min^–1^, respectively, which is higher than microwave-assisted UV (MDEL)/TiO_2_/K_2_S_2_O_8_ (0.0216 min^–1^),^52^ Cu_2_S–Cu–TiO_2_ mesoporous carbon composites (0.0173 min^–1^),^53^ and Fe-sand (0.024 min^–1^)^54^ treatments. Notably, a previous work using CuCa HDS^19^ as a CWAO catalyst in MO degradation gave a MO decolorization rate of 0.87 min^–1^ which is close to that obtained from CuCa_HDS_QES_750. Nevertheless, higher MO decolorization rates can be achieved by using CuCa_HDS_DES_850. To our knowledge, the most active Cu_2_(OH)_3_NO_3_/ZnO^20^ gave the highest MO decolorization rate (3.90 min^–1^), suggesting that the activity of HDS materials depends on the metal oxide precursor used. Based on the literature, for the medium range of initial MO concentrations, Ag/CdS@Pr/TiO_2_^55^ and Ag–AgBr/BiVO_4_/graphene aerogel,^56^ respectively, were highly active in MO decolorization giving MO decolorization rates of 0.044 and 0.1097 min^–1^. However, those catalysts showed much lower MO decolorization rates than those of CuCa_HDS_DES_850 and CuCa_HDS_QES_750. Therefore, eggshell-derived CuCa HDS materials are effective for use in treatments of highly concentrated MO(aq), giving high color and COD removal efficiencies and high MO decolorization rates. The findings from this work highlight the low-cost preparation method of the CuCa HDS materials by using calcined eggshells as raw materials instead of nonrenewable calcined limestone. The calcination temperature of 750 °C or above can produce bio-CaO raw materials that can be further used to prepare highly active CuCa HDS, a promising material for effective dye removal. It should also be emphasized that the CuCa HDS treatments were performed under ambient conditions to achieve the effective removal of MO and MO degradation products. Compared with other CWAO processes or other remediation methods, MO removal does not require additional oxidizing agents, high temperature and pressure, or any other forms of energy input to achieve the best performance.

## 4 Conclusions

In summary, both limestones and eggshells require burning processes in order to produce quicklime or biobased CaO, respectively. However, in contrast to limestones, eggshells are potentially renewable and more environmentally friendly. Based on the literature and the results from this work, biobased CaO derived from the calcination of waste eggshells from different sources (hen eggshells, QESs, and DESs) can be used to produce CuCa HDS, the material that induces oxidative MO decolorization in aqueous solutions. Consequently, at least three types of eggshells (from duck, quail, and chicken) exhibited high CuCa HDS activity, indicating that eggshell waste can be collected for subsequent processing without sorting. Intercalation reactions are also responsible for effective MO removal via interlayered ion exchanges. To obtain a suitable precursor for preparing such effective CuCa HDS materials, calcination temperatures of 750 °C or higher should be used. The eggshell-derived CuCa HDS materials could be reused for three consecutive cycles without the requirement of regeneration, and the color removal efficiency was greater than 90% in the third run. An acid wash method can be used to regenerate the ineffective HDS after the fourth cycle. Comparative pseudo-first-order reaction rates of MO decolorization by various materials suggested that the eggshell-derived CuCa HDS materials are highly effective at high initial MO concentrations (highly polluted water, COD > 500 mg O_2_/L).^19^ Thus, HDS treatments are one of the most promising methods for pretreatment of highly toxic wastewater that typically makes biological treatments ineffective. Further investigation on utilizing eggshell-derived CaO in preparing other technological materials will significantly boost confidence in large-scale materials processing and eggshell waste recycling.

## Supporting Information Available

Additional experimental data (XRD, TGA, DRS profiles, and SEM images of relevant samples) and literature summary (PDF)
