# Supplementary material for: Eggshell-Derived Copper Calcium Hydroxy Double Salts and Their Activity for Treatment of Highly Polluted Wastewater
Source: ACS Omega. 2023 Nov 29;8(49):46663–75. doi: 10.1021/acsomega.3c05758 (PMC10719995; doi:10.1021/acsomega.3c05758)
Supplement: Supplementary file 1 — ao3c05758_si_001.pdf [file ao3c05758_si_001.pdf]

Supplementary Information

Eggshell-derived Copper Calcium Hydroxy Double Salts and their Activity for Treatment of Highly Polluted Wastewater

Yiping Han,[a,b] Jirawat Trakulmututa,[b,□] Taweechai Amornsakchai, [a,b] Supakorn Boonyuen,[c] Nicha Prigyai,[b,#] Siwaporn Meejoo Smith [a,b,*]

[a.] Natural Resources and Waste Module, Department of Chemistry, Faculty of Science, Mahidol University, Rama VI Rd, Rajathewi, 10400, Thailand.

[b.] Center of Sustainable Energy and Green Materials, Faculty of Science, Mahidol University, Salaya, Nakorn Pathom, 73170, Thailand.

[c.] Department of Chemistry, Faculty of Science and Technology, Thammasat University, Paholyothin, Klong-Luang, Pathumthani 12120, Thailand.

* Corresponding author: siwaporn.smi@mahidol.edu; Tel.: (+66) 93 593 9449 Fax: (+66) 2 354 7151

□ Current address: Department of Earth Resources Engineering, Faculty of Engineering, Kyushu University, 744 Motooka, Nishi-ku, Fukuoka, 819-0395, Japan

# Current address: Nuclear Technology Research and Development Center, Thailand Institute of Nuclear Technology (Public Organization), Saimoon, Ongkarak, Nakhon Nayok 26120, Thailand.

Table of Contents

		Page
**Figure S1.**	a) Powder X-ray diffraction profile and b) thermogravimetric plot for the CuCa HDS D_850	S3
**Figure S2.**	Diffuse reflectance spectroscopic result and b) Tauc plot for the CuCa HDS D850	S4
**Figure S3.**	SEM morphology of uncalcined and calcined eggshells (DES, and QES series)	S5
**Figure S4.**	Powder X-ray diffraction profiles for the acid washed CuCa HDS D_850 material (Spent, 4th cycle) and that in the 5th cycle at a) low- and b) high-angle ranges. From the first to the fifth cycle, no changes in the structure of spent CuCa HDS D_850 material were observed. The diffraction peaks corresponding to MO were more evident, with higher intensity, in the 5th cycle.	S6
**Table S1.**	Comparison of various materials used to degrade aqueous methyl orange dye	S7

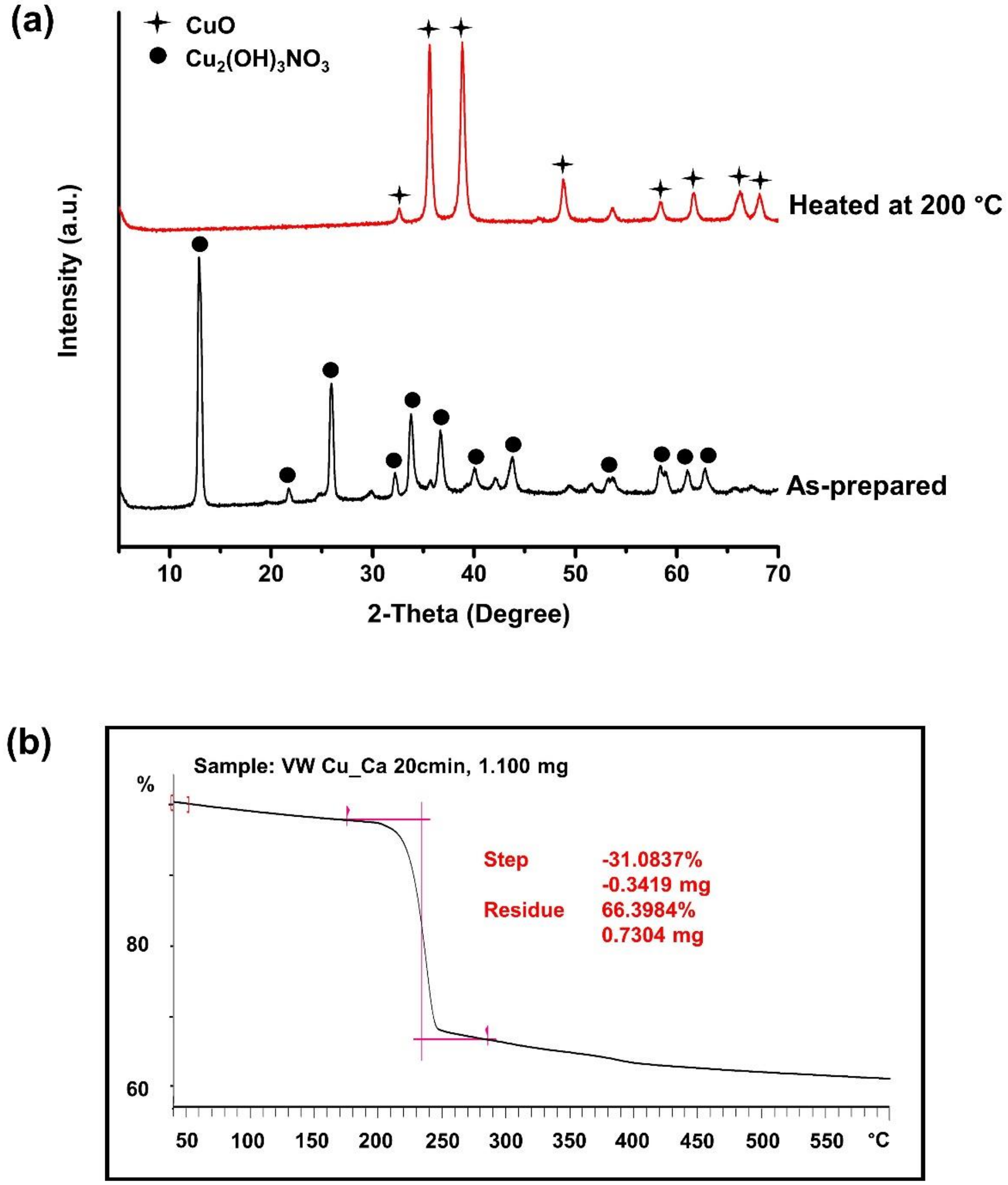

Figure S1. a) Powder X-ray diffraction profile and b) thermogravimetric plot for the CuCa HDS D_850

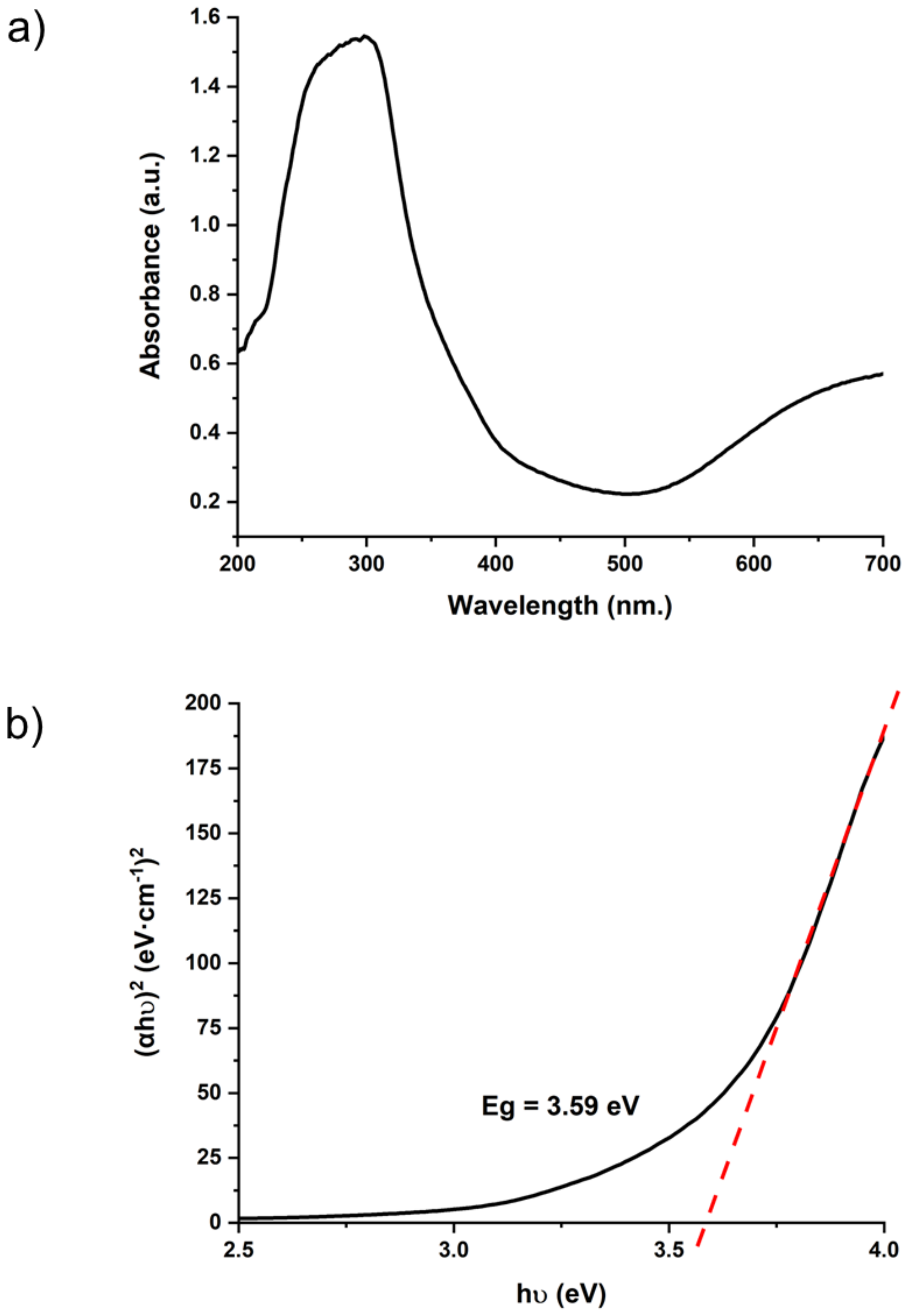

Figure S2. a) Diffuse reflectance spectroscopic result and b) Tauc plot for the CuCa HDS D850. The CuCa HDS is a wide band gap material. Their activity for the dye removal was not related to their energy bandgap (3.59) or photocatalytic activity, as they are active either in the dark or under ambient light irradiation.

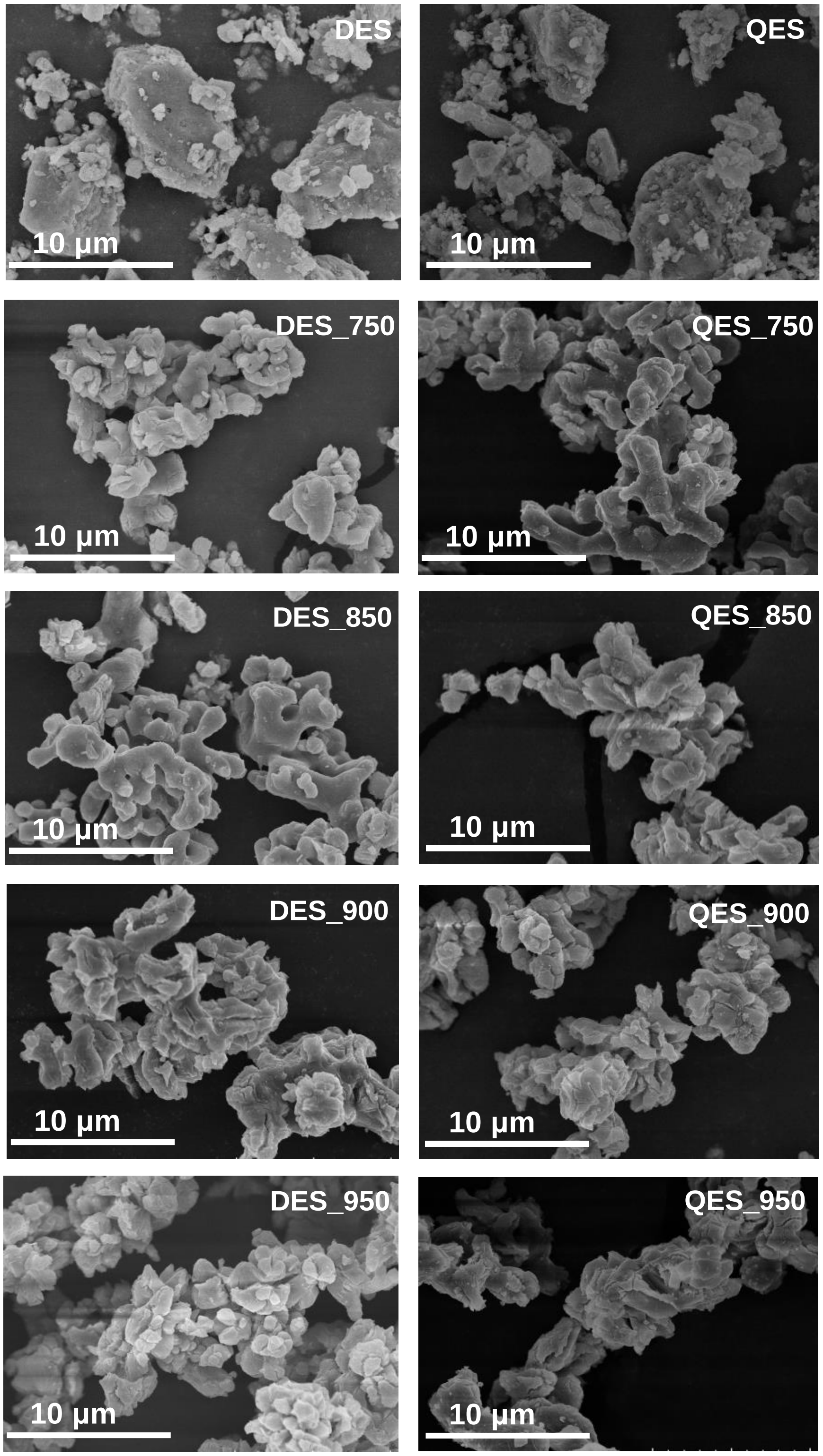

Figure S3. SEM morphology of uncalcined and calcined eggshells (DES, and QES series) in 5000x magnifications.

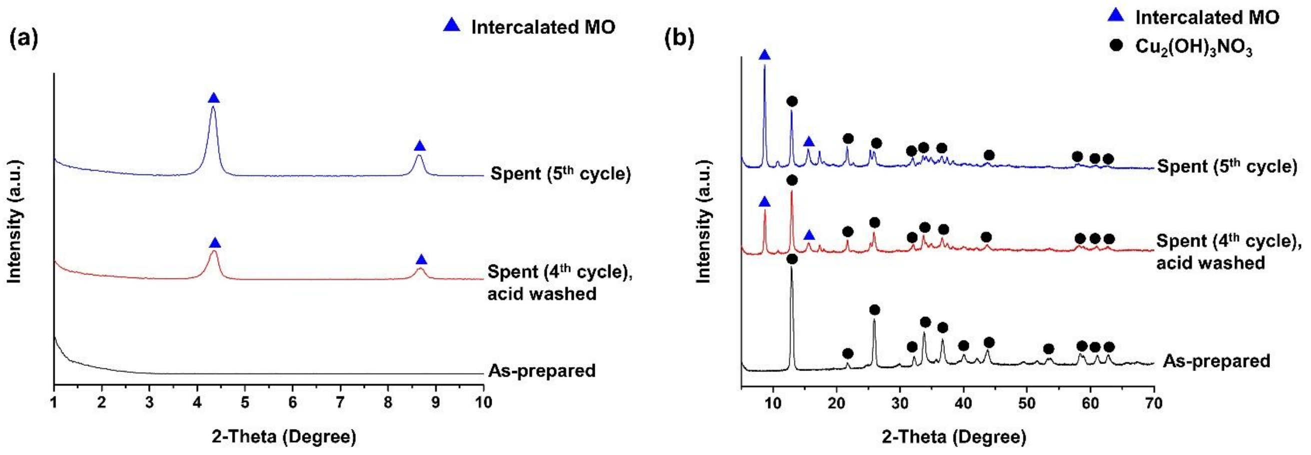

Figure S4. Powder X-ray diffraction profiles for the acid washed CuCa HDS D_850 material (Spent, 4th cycle) and that in the 5th cycle at a) low- and b) high-angle ranges. From the first to the fifth cycle, no changes in the structure of spent CuCa HDS D_850 material were observed. The diffraction peaks corresponding to MO were more evident, with higher intensity, in the 5th cycle.

Table S1. Comparison of various materials used to degrade aqueous methyl orange dye.

Catalyst	Type	Additives	[MO] (ppm)	Dosage (g/L)	Time (min)	Reaction conditions	Removal efficiency (%)	Reaction rate (min^{-1})	Kinetic model	Ref.
Microwave-assisted UV (MDEL)/TiO_2/ $K_2S_2O_8$	Microwave-assisted UV/TiO_2/ $K_2S_2O_8$ oxidation process	$K_2S_2O_8$, O_2 (g)	28,543	0.2025	60	Microwave-assisted UV(MDEL)/TiO_2/$K_2S_2O_8$ hybrid system (continuous flow)	80.0	0.0216	Pseudo-first order	[1]
Cu-Fe-La/ γ-Al_2O_3	Catalytic wet air oxidation (CWAO)	O_2 (g)	3,000	4	120	T= 200 ℃, 2.0 MPa (O_2) (stirred-batch reactions)	94.1	-	-	[2]
CuO/γ-Al_2O_3	Catalytic wet air oxidation (CWAO)	none	1,000	27.71	60	pH= 3, T= 80 ℃, atmosph- eric pressure (stirred-batch reactions)	100	-	-	[3]
g-C_3N_4/Au	Catalytic reduction	$NaBH_4$	982	0.4	8	Ambient conditions (stirred-batch reactions)	Nearly 100%	-	-	[4]
CuCa HDS_DES_850	Catalytic wet air oxidation (CWAO)	none	500	2	5	Ambient conditions (stirred-batch reactions)	100.0	1.20	Pseudo-first order	This work

Catalyst	Type	Additives	[MO] (ppm)	Dosage (g/L)	Time (min)	Reaction conditions	Removal efficiency (%)	Reaction rate (min^{-1})	Kinetic model	Ref.
CuCa HDS_QES_750	Catalytic wet air oxidation (CWAO)	none	500	2	5	Ambient conditions (stirred-batch reactions)	100.0	0.85	Pseudo-first order	This work
$Cu_2(OH)_3NO_3$/ZnO	Catalytic wet air oxidation (CWAO)	none	500	3	5	Ambient conditions (stirred-batch reactions)	99.8	3.90	Pseudo-first order	[5]
Cu-Ca HDS	Catalytic wet air oxidation (CWAO)	none	500	2	10	Ambient conditions (stirred-batch reactions)	100	0.87	Pseudo-first order	[6]
Fe_2O_3-CeO_2-TiO_2/γ-Al_2O_3	Catalytic wet air oxidation (CWAO)	none	500	30	150	T= 25 ℃, atmospheric pressure, 400 ml/min air flow rate (stirred-batch reactions)	98.09	-	-	[7]
Cu_2S-Cu-TiO_2 mesoporous carbon composites	Photocatalytic degradation	none	300	0.6	120	Stirred in the dark for 60 min, irradiated by a high-pressure Na lamp (stirred-batch reactions)	94.0	0.0173	Pseudo-first order	[8]

Catalyst	Type	Additives	[MO] (ppm)	Dosage (g/L)	Time (min)	Reaction conditions	Removal efficiency (%)	Reaction rate (min^{-1})	Kinetic model	Ref.
Fe-sand	Photo-Fenton oxidation	H_2O_2	150	1.5	60	Irradiated by a high-pressure Hg lamp (125W, λ_{max} = 365 nm), pH 2.5 (stirred-batch reactions)	100	0.024	Pseudo-first order	[9]
NiAlCe LDH modified with DBD plasma (75V/20min)	Photocatalytic degradation	none	80	1	36	Stirred in dark conditions for 30-45 min, under ultraviolet light irradiation of mercury lamp (500W) (stirred-batch reactions)	Nearly 100%	-	-	[10]
CuO/CeO_2	Microwave assisted catalytic degradation in the present of H_2O_2	H_2O_2	50	8	7	Microwave apparatus: 380W/ 2450 MHz, 7min, pH= 6-7	85.2	-	-	[11]
Ag:CdS@Pr:TiO_2	Photocatalytic degradation and Catalytic reduction	$NaBH_4$	32	0.032	30	Ambient conditions, visible light (stirred-batch reactions)	98.0	0.044	Pseudo-first order	[12]

Catalyst	Type	Additives	[MO] (ppm)	Dosage (g/L)	Time (min)	Reaction conditions	Removal efficiency (%)	Reaction rate (min^{-1})	Kinetic model	Ref.
Graphene aerogel/bismuth oxyiodide (GA/BiOI)	Photocatalytic degradation	none	20	0.4	120	Stirred in the dark for 30 min, irradiated by Xe lamp (5 × 55 W) at an intensity of 190 mW/cm^2 (stirred-batch reactions)	93.1	0.0165	Pseudo-first order	[13]
[FemIL@SiO_2 @Mag]$_2$$MoO_4$	Photocatalytic degradation	H_2O_2	20	1	30	Photoreactor open in air, stirred in the dark for 30 min, irradiated by high-pressure Hg lamp (250 W, λ_{max} = 365 nm) (stirred-batch reactions)	99.0	0.007997	Pseudo-first order	[14]
CuAl-LDH	Heterogeneous Fenton-like degradation	H_2O_2	20	0.4	10	Heated in a water bath at T= 40 °C, under atmospheric pressure (stirred-batch reactions)	100	-	-	[15]
MnO_2/CeO_2	Catalytic ultrasonic degradation	none	20	1	10	pH= 2.8, ultrasonic irradiation 650 W/24 kHz with 1.3 W/ml ultrasonic density, 10 min (stirred-batch reactions)	90	-	-	[16]

Catalyst	Type	Additives	[MO] (ppm)	Dosage (g/L)	Time (min)	Reaction conditions	Removal efficiency (%)	Reaction rate (min^{-1})	Kinetic model	Ref.
Cu-doped ZnO nanorods	Photocatalytic degradation	none	13	0.3	120	Sonicated for 3 min, then kept in the dark for 15 min, irradiated under the sun illumination (0.1W /cm^2) (stirred-batch reactions)	99.0	0.024	Pseudo-first order	[17]
GW-ZnMCPPc (Zinc phthalocyanines conjugated with glass wool)	Photocatalytic degradation	none	10.8	3.3	720	Illuminated with Modu-light 690 nm laser (irradiance of 1.0 W/cm2) at 15 min (stirred-batch reactions)	100	0.0049	Pseudo-first order	[18]
Mesoporous-assembled $SrTiO_3$ nanocrystal	Photo-Fenton oxidation	none	10	0.005	60	Irradiated by UV light (15W, 365 nm) at room temperature (stirred-batch reactions)	100	0.013	Pseudo-first order	[19]
Ag/AgCl/CdSnO_3.3H_2O	Photocatalytic degradation	none	10	1	40	Stirred in darkness for 30 min, irradiated by Xe lamp (300 W, λ > 420 nm) (batch reactions)	94.0	0.07277	Pseudo-first order	[20]

Catalyst	Type	Additives	[MO] (ppm)	Dosage (g/L)	Time (min)	Reaction conditions	Removal efficiency (%)	Reaction rate (min^{-1})	Kinetic model	Ref.
$CuI/g-C_3N_4$	Photocatalytic degradation	none	10	1	80	Stirred in the dark for 30 min, under UV (Osram ULTRA-VITALUX 400 W) (batch reactions)	98.0	0.0298	Pseudo-first order	[21]
$Sn-WO_3/g-C_3N_4$	Photocatalytic degradation	none	10	0.4	120	Sonicated for 1 hour in the dark, under halogen lamp (150 W) as visible light source (stirred-batch reactions)	87.0	0.0142	Pseudo-first order	[22]
Ag-AgBr $/BiVO_4/$ graphene aerogel	Photocatalytic degradation	none	10	1	24	Immersed into MO solution for 30 min, under Xe lamp (500 W, $\lambda > 420$ nm) (batch reactions)	93.9	0.1097	Pseudo-first order	[23]
$Ag_2Mo_{1-x}W_xO_4$ (x = 0.50 mol%)	Photocatalytic degradation	none	0.2	1	150	Stirred for 5 min, pH 5, irradiated by UVC lamps (15W) (batch reactions)	45.0	0.00585	Pseudo-first order	[24]
Cu-doped Bi_2S_3	Photocatalytic degradation	none	0.1	0.1	90	Stirred in the dark cupboard for 30 min, irradiated by halogen lamp for 1–2 hours (batch reactions)	94.0	0.0256	Pseudo-first order	[25]

References

1 Lee, H.; Park, Y. K.; Kim, S. J.; Kim, B. H.; Yoon, H. S.; Jung, S.-C. Rapid Degradation of Methyl Orange Using Hybrid Advanced Oxidation Process and Its Synergistic Effect. *J. Ind. Eng. Chem.* **2016**, *35*, 205–210.

2 Zhang, Y.; Peng, F.; Zhou, Y. Structure, Characterization, and Dynamic Performance of a Wet Air Oxidation Catalyst Cu–Fe–La/γ-Al2O3. *Chin. J. Chem. Eng.* **2016**, *24*, 1171–1177.

3 Hua, L.; Ma, H.; Zhang, L. Degradation Process Analysis of the Azo Dyes by Catalytic Wet Air Oxidation with Catalyst $CuO/\gamma\text{-}Al_2O_3$. *Chemosphere.* **2013**, *90*, 143–149.

4 Nasri, A.; Jaleh, B.; Nezafat, Z.; Nasrollahzadeh, M.; Azizian, S.; Jang, H. W.; Shokouhimehr, M. Fabrication of $G\text{-}C_3N_4/Au$ Nanocomposite Using Laser Ablation and Its Application as an Effective Catalyst in the Reduction of Organic Pollutants in Water. *Ceram. Int.* **2021**, *47*, 3565–3572.

5 Srikhaow, A.; Smith, S. M. Preparation of $Cu_2(OH)_3NO_3/ZnO$, a Novel Catalyst for Methyl Orange Oxidation under Ambient Conditions. *Appl. Catal. B: Environ.* **2013**, *130–131*, 84–92.

6 Weeramonkhonlert, V.; Srikhaow, A.; Smith, S. M. Formation of Copper Hydroxy Double Salts Derived from Metal Oxides and Their Catalytic Activity in Degradation of Methyl Orange. *Ceram. Int.* **2019**, *45*, 993–1000.

7 Liu, Y.; Sun, D. Development of $Fe_2O_3\text{-}CeO_2\text{-}TiO_2/\gamma\text{-}Al_2O_3$ as Catalyst for Catalytic Wet Air Oxidation of Methyl Orange Azo Dye under Room Condition. *Appl. Catal. B.* **2007**, *72*, 205–211.

8 Zhang, L.; Zhao, Y.; Zhong, L.; Wang, Y.; Chai, S.; Yang, T.; Han, X. $Cu_2S\text{-}Cu\text{-}TiO_2$ Mesoporous Carbon Composites for the Degradation of High Concentration of Methyl Orange under Visible Light. *Appl. Surf. Sci.* **2017**, *422*, 1093–1101.

9 Omri, A.; Hamza, W.; Benzina, M. Photo-Fenton Oxidation and Mineralization of Methyl Orange Using Fe-Sand as Effective Heterogeneous Catalyst. *J. Photochem. Photobiol., A.* **2020**, *393*, 112444.

10 Tao, X.; Han, Y.; Sun, C.; Huang, L.; Xu, D. Plasma Modification of NiAlCe–LDH as Improved Photocatalyst for Organic Dye Wastewater Degradation. *Appl. Clay Sci.* **2019**, *172*, 75–79.

11 Xu, D.; Cheng, F.; Lu, Q.; Dai, P. Microwave Enhanced Catalytic Degradation of Methyl Orange in Aqueous Solution over CuO/CeO_2 Catalyst in the Absence and Presence of H2O2. *Ind. Eng. Chem. Res.* **2014**, *53*, 2625–2632.

12 Singh, A.; Ahmed, A.; Sharma, A.; Sharma, C.; Paul, S.; Khosla, A.; Gupta, V.; Arya, S. Promising Photocatalytic Degradation of Methyl Orange Dye via Sol-Gel Synthesized Ag–CdS@Pr-TiO_2 Core/Shell Nanoparticles. *Phys. B: Condens.* **2021**, *616*, 413121.

13 Arumugam, M.; Seralathan, K. K.; Praserthdam, S.; Tahir, M.; Praserthdam, P. Synthesis of Novel Graphene Aerogel Encapsulated Bismuth Oxyiodide Composite towards Effective Removal of Methyl Orange Azo-Dye under Visible Light. *Chemosphere.* **2022**, *303*, 135121.

14 Naikwade, A. G.; Jagadale, M. B.; Kale, D. P.; Gophane, A. D.; Garadkar, K. M.; Rashinkar, G. S. Photocatalytic Degradation of Methyl Orange by Magnetically Retrievable Supported Ionic Liquid Phase Photocatalyst. *ACS Omega.* **2020**, *5*, 131–144.

15 Li, J.; Zhang, S.; Chen, Y.; Liu, T.; Liu, C.; Zhang, X.; Yi, M.; Chu, Z.; Han, X. A Novel Three-Dimensional Hierarchical CuAl Layered Double Hydroxide with Excellent Catalytic Activity for Degradation of Methyl Orange. *RSC Adv.* **2017**, *7*, 29051–29057.

16 Zhao, H.; Zhang, G.; Chong, S.; Zhang, N.; Liu, Y. MnO_2/CeO_2 for Catalytic Ultrasonic Decolorization of Methyl Orange: Process Parameters and Mechanisms. *Ultrason. Sonochem.* **2015**, *27*, 474–479.

17 Perillo, P. M.; Atia, M. N. Solar-Assisted Photodegradation of Methyl Orange Using Cu-Doped ZnO Nanorods. *Mater. Today Commun.* **2018**, *17*, 252–258.

18 Sindelo, A.; Britton, J.; Lanterna, A. E.; Scaiano, J. C.; Nyokong, T. Decoration of Glass Wool with Zinc (II) Phthalocyanine for the Photocatalytic Transformation of Methyl Orange. *J. Photochem. Photobiol., A.* **2022**, *432*, 114127.

19 Puangpetch, T.; Sreethawong, T.; Yoshikawa, S.; Chavadej, S. Synthesis and Photocatalytic Activity in Methyl Orange Degradation of Mesoporous-Assembled $SrTiO_3$ Nanocrystals Prepared by Sol–Gel Method with the Aid of Structure-Directing Surfactant. *J. Mol. Catal. A. Chem.* **2008**, *287*, 70–79.

20 Yang, S.-F.; Niu, C. G.; Huang, D. W.; Zhang, H.; Zeng, G. M. Ag/AgCl Nanoparticles-Modified $CdSnO_3{\cdot}3H_2O$ Nanocubes Photocatalyst for the Degradation of Methyl Orange and Antibiotics under Visible Light Irradiation. *J. Colloid Interface Sci.* **2017**, *505*, 96–104.

21 Ghanbari, M.; Salavati-Niasari, M. Copper Iodide Decorated Graphitic Carbon Nitride Sheets with Enhanced Visible-Light Response for Photocatalytic Organic Pollutant Removal and Antibacterial Activities. *Ecotoxicol. Environ. Saf.* **2021**, 208, 111712.

22 Ahmed, K. E.; Kuo, D. H.; Zeleke, M. A.; Zelekew, O. A.; Abay, A. K. Synthesis of Sn-WO3/g-C3N4 Composites with Surface Activated Oxygen for Visible Light Degradation of Dyes. *J. Photochem. Photobiol., A.* **2019**, *369*, 133–141.

23 Lin, L.; Xie, Q.; Zhang, M.; Liu, C.; Zhang, Y.; Wang, G.; Zou, P.; Zeng, J.; Chen, H.; Zhao, M. Construction of Z-Scheme Ag-AgBr/$BiVO_4$/Graphene Aerogel with Enhanced Photocatalytic Degradation and Antibacterial Activities. *Colloids Surf. A: Physicochem. Eng. Asp.* **2020**, *601*, 124978.

24 Andrade Neto, N. F.; Lima, A. B.; Bomio, M. R. D.; Motta, F. V. Microwave-Assisted Hydrothermal Synthesis of Ag_2Mo_1-$xWxO_4$ (x = 0, 0.25, 0.50, 0.75 and 1 Mol%) Heterostructures for Enhanced Photocatalytic Degradation of Organic Dyes. *J. Alloys Compd.* **2020**, *844*, 156077.

25 Onwudiwe, D. C.; Nkwe, V. M.; Olatunde, O. C.; Ferjani, H. Graphitic Carbon Nitride Functionalized with Cu-Doped Bi_2S_3 as a Heterostructure Photocatalyst for the Visible Light Degradation of Methyl Orange. *Ceram. Int.* **2023**, *49*, 19451–19462.
